# Red Photoactivatable Genetic Optical-Indicators

**DOI:** 10.3389/fncel.2020.00113

**Published:** 2020-05-28

**Authors:** Wessal Hussein, Shai Berlin

**Affiliations:** Department of Neuroscience, The Ruth and Bruce Rappaport Faculty of Medicine, Technion – Israel Institute of Technology, Haifa, Israel

**Keywords:** photoactivatable, GECI genetically encoded Ca^2+^ indicator, calcium, red fluorescent protein, optical tools

## Abstract

Emerging genetically-encoded Ca^2+^-indicators (GECIs) are intensiometric reporters that increase in fluorescence when bound to Ca^2+^; highly suited for studying calcium-signaling in many cell types, notably neurons. Today, major efforts are devoted toward optimizing red-emitting [red fluorescent protein (RFP)-based] GECIs (R-GECI), as these provide several advantages over GFP-based reporters, for instance, increased imaging depth, reduced photodamage by longer imaging wavelengths and, in principle, are better suited for use with prevalent blue-absorbing optogenetic tools (e.g., channelrhodopsin). However, excessive fluorescence from intersecting neighboring cells in very dense tissues, notably the brain, hinders the ability to collect signals from single cells and their processes. This challenge can be addressed by photoactivatable (PA) fluorescent proteins that can be rendered fluorescent *on demand* by user-defined targeted light. This allows activation and, thereby, collection of fluorescent signals exclusively from desired cells and their processes, while leaving all neighboring cells in the dark (i.e., non-fluorescent). Nevertheless, there are no PA R-GECIs. Here, we sought to develop PA-R-GECIs. To do so, we initially explored a recently discovered phenomenon of Ca^2+^-independent increases in fluorescence (i.e., artifacts) in an emerging R-GECI, which has led us to rationally engineer several functional PA-R-GECIs. We also take advantage of our findings to quickly engineer a novel PA-RFP, namely, PA-mRuby3.

## Introduction

There is a growing interest in red-shifted genetically-encoded Ca^2+^-indicators (R-GECI) as these are activated by longer and less cytotoxic wavelengths, provide deeper imaging depths and, importantly, ease on concomitant use with common optogenetic or synthetic optogenetic tools (Yizhar et al., 2011; Berlin and Isacoff, 2017; Piatkevich et al., 2019). R-GECIs are therefore ideal for *all optical* neural interrogation *in vivo* (Emiliani et al., 2015). However, when studying cellular and subcellular Ca^2+^-dynamics *in vivo*, it is often difficult to monitor Ca^2+^-signals from single neuronal somata owing to background fluorescence, namely, fluorescence emanating from adjacent cells and processes located next to, above, or below the desired cell. Background fluorescence also makes it very challenging for tracing and assigning processes to defined somata. These limitations have previously motivated us to design green photoactivatable (PA)-GECIs (PA-GCaMP; Berlin et al., 2015). Akin to PA-GFP (e.g., Patterson and Lippincott-Schwartz, 2002), the capability to control the basal fluorescence of GCaMP in single cells by light enables users to optically label (“highlight”) selected cells with very high spatiotemporal resolution (Patterson and Lippincott-Schwartz, 2002; Piatkevich et al., 2013). This robustly improves the signal-to-noise ratio and permits subsequent collection of signals (i.e., Ca^2+^-dependent changes in fluorescence) exclusively from highlighted cells and their processes with higher certainty and ease. Whereas other methods can address some of these challenges by sparsely labeling single neurons (Lee et al., 2019), optical highlighting of cells provides one major advantage over these, namely, granting users access to all cells in the preparation (for instance, in a transgenic model), rather than restricting the user to a small and stochastic population of cells. Moreover, photoactivation can be performed sequentially thereby allowing to monitor many cells in the same preparation, including adjacent ones.

**FIGURE 1:**
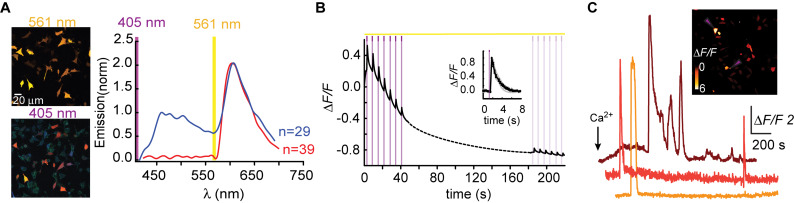
R-GECO1 is blue and red-emitting fluorescent sensor. **(A)** Emission spectra collected after excitation with 405 nm (magenta bar, blue plot) and 561 nm (yellow bar, red plot). Insets—Micrographs of HeLa cells expressing R-GECO1 and illuminated by 561 nm (top) or 405 nm (bottom). Note the bright red fluorescence following 405 nm (e.g., white arrows). **(B)** R-GECO1 exhibiting transient photoconversion during intervals of near UV light irradiation (405 nm). **(C)** Representative traces of Ca^2+^-activity induced by application of excessive Ca^2+^ (see section “Materials and Methods”).

Of the handful photoconvertible (Hoi et al., 2012; Fosque et al., 2015; Zolnik et al., 2017) and PA-sensors (Matsuda et al., 2013; Berlin et al., 2015; Lee et al., 2019), *none* are of single red-emission (see Walia et al., 2018). We therefore sought to develop a single-emission red-PA-GECI. Sequence analysis of extant PA-red fluorescent proteins (RFPs) reveals that the most optimized R-GECI variants are based on RFPs to which there are no PA versions. Moreover, unlike green PA-FPs, there are no shared set of mutations that can be easily transferred among different members of the RFP superfamily to endow them with PA features. This likely stems from the fact that the structural basis for photoactivation in PA-RFPs is less understood (and likely less conserved) than in members of the GFP superfamily (Lukyanov et al., 2005; Piatkevich and Verkhusha, 2010). Consequently, PA-RFPs are almost exclusively engineered via random mutagenesis schemes. To rationally design PA-R-GECI, we began by focusing on R-GECO1 (red-genetically encoded Ca^2+^-indicator for optical imaging; Zhao et al., 2011). R-GECO1 is a potent red-GECI with very large responses (Δ*F/F)*, wide dynamic-range and is suitable for two-photon imaging, to name a few (Zhao et al., 2011; Dana et al., 2016). Importantly, R-GECO1 is based on mApple; a unique FP that exhibits intrinsic and transient photoconvertible behavior (Shaner et al., 2008). The functional outcome of this behavior is the appearance of transient light-dependent—and Ca^2+^-independent—increases in fluorescence (here denoted artifacts) when illuminated with near-UV-to-green light (Wu et al., 2013 and see Figure 1). These artifacts display similar features (size and kinetics) as *bona fide* Ca^2+^-dependent responses, making it very hard to distinguish between them. This limitation thereby complicates the use of R-GECO1 with near-UV-to-green light activated optogenetic tools (Shaner et al., 2008; Akerboom et al., 2013; Wu et al., 2013; Berlin et al., 2016; Piatkevich et al., 2019). Nevertheless, here we benefitted from this behavior for the development of PA Red-GECIs and a new PA-RFP.

## Results

### Red Ca^2+^-Indicators Display Blue Emission and Ca^2+^-Independent Increases in Fluorescence

We, and others, have recently noted that R-GECO1 undergoes reversible photoactivation by blue-shifted wavelengths (e.g., Wu et al., 2013). Unfortunately, the mechanism of this phenomenon remains poorly understood (speculated to range from light-induced deprotonation, formation of new chromophore-type to chromophore isomerization) (Akerboom et al., 2013; Wu et al., 2013; Berlin et al., 2016). To try and harness this behavior for generating a PA R-GECI, we first turned to characterize this behavior in our system and cells. Cells expressing R-GECO1 and illuminated by 561 nm exhibit bright red fluorescence (Figure 1A, top micrograph and red plot, 561 nm excitation—yellow bar). When short and intermittent bouts of 405 nm illumination are applied, robust and transient increases in red fluorescence are obtained (i.e., artifacts); highly reminiscent of *bona fide* Ca^2+^-dependent increases in fluorescence (Figures 1B,C). These observations suggest that R-GECO1 directly absorbs 405 nm. Indeed, 405 nm excitation elicits two emission peaks of blue and red fluorescence, λ_peak_ = 460 and 600 nm, respectively (Figure 1A, bottom micrograph—note the apparent blue and red cells; and blue plot, 405 nm excitation—magenta bar). While R-GECO1 has been shown to absorb near-UV light (Akerboom et al., 2013; Wu et al., 2013), to the best of our knowledge, the description of its emission(s) in response to shorter wavelengths has not been reported. R-GECO1’s emissions of blue and red spectra suggested to us the presence of two populations of R-GECO in cells; each with a different maturation state of its chromophore. In fact, most (if not all) RFPs begin as blue-emitters before their methionine-tyrosine-glycine (MYG) chromophore develops into its final red-emitting form (see full sequence, Supplementary Figure 1, red box) (Subach et al., 2008; Shcherbakova et al., 2012). Thus, the near-UV induced artifacts may result from several mechanisms, such as the direct excitation of the red-chromophore, light-induced isomerization, or, less likely, instantaneous maturation of the blue-chromophore into red (Wu et al., 2013 and see below).

### Generation of Photoactivatable Red Ca^2+^-Indicators

**FIGURE 2:**
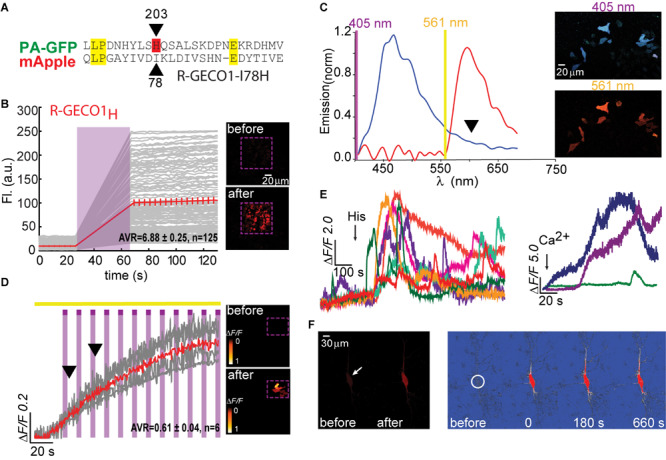
Photoactivatable R-GECO1_H_. **(A)** Partial sequence alignment between PA-GFP and mApple. Black arrow indicates T203H position and its parallel I78 in mApple. **(B)** R-GECO1_H_ in HEK293T cells during prolonged photoactivation with 100% of 405 nm at ∼1.7 mW (magenta bar). Insets—HEK cells imaged by 561 nm before (top) and after (bottom) photoactivation (magenta dashed square). **(C)** Emission spectra collected before photoactivation by use of 405 nm (blue plot) and 561 nm (red plot). Insets—Micrographs of HeLa cells expressing R-GECO1_H_ and illuminated by 405 nm (top) or 561 nm (bottom). Note the lack of red fluorescence following 405 nm excitation. **(D)** Stable photoactivation of R-GECO1_H_ in HeLa cells. Cells imaged by 561 nm (yellow bar) are intermittently excited by short (1 s) bouts of 405 nm (magenta bars) exhibit stable increase in Ca^2+^-independent fluorescence, until reaching a plateau. Insets—HeLa cells imaged by 561 nm before (top) and after (bottom) photoactivation. **(E)** R-GECO1_H_ is functional as a Ca^2+^-probe. Representative traces showing Ca^2+^-activity following the application of histamine (15 μM, left) and Ca^2+^ (+50 μM, right). **(F)** Hippocampal neuron before and after somatic photoactivation (left micrographs). Note the low, albeit noticeable, basal fluorescence (white arrow). Right images (Δ*F/F*) show spread of fluorescence throughout the dendritic arbor over 600 s, following somatic photoactivation of R-GECO1_H_ (white circle).

To benefit from the artifactual behavior of R-GECO, we first set out to locate residues within R-GECO1 (i.e., mApple) that would be amenable to rational mutagenesis so as to convert the transient photoconversion into stable photoactivation. We first examined the sequences of various PA-FPs, explicitly PA-GFP, PA-mCherry, and PA-TagRFP (Patterson and Lippincott-Schwartz, 2002; Lukyanov et al., 2005), in addition to sequences of RFPs that serve as templates for R-GECIs, namely, mPlum (backbone of R-GECO1.2; Wu et al., 2013) and mRuby (backbone of RCaMP1h and jRCaMP; Akerboom et al., 2013; Dana et al., 2016; Supplementary Figure 1). Interestingly, PA-GFP and PA-RFPs show no obvious shared pattern of mutations, except for the involvement of the well-recognized polar residue essential for the PA behavior of PA-GFP (Figure 2A; black arrowhead and Supplementary Figure 1, black arrowhead; Patterson and Lippincott-Schwartz, 2002). Notably, whereas the 203rd residue is essential for the photoactivation of both red and green FPs, a histidine in that position does not seem to be essential for RFPs. For instance, PA-mCherry1, 2, and -3 (Subach et al., 2009), and PA-TagRFP (Subach et al., 2010) are PA but with an arginine substitution instead.

We therefore deemed it worthy to focus on this residue in R-GECO1 (mApple; residue I78) (see section “Materials and Methods” and primer list). Importantly, whereas PA-GFP required two additional mutations for photoactivation, specifically phenylalanine preceding the chromophore and a serine within it (F64 and S65; GFP numbering; Patterson and Lippincott-Schwartz, 2002), most PA and non-PA RFPs examined (including mApple of R-GECO1) retain the original chromophore (MYG) and the preceding hydrophobic phenylalanine (Supplementary Figure 1, red box). The only exception in our screen was mPlum (template for R-GECO1.2; Wu et al., 2013; and see below). mPlum does not bear the F64 residue, rather I64 (Supplementary Figure 1, blue circle). However, these two amino acids are similarly hydrophobic and therefore we did not deem it necessary to substitute it or any additional residues.

Based on the above-mentioned, we naturally turned to firstly engineer R-GECO1-I78H; R-GECO1_H_ (Figure 2). When expressed in HEK293T cells, very low basal fluorescence (prior Ca^2+^ addition or photoactivation) could be detected when imaged at 561 nm (Figure 2B, 0–20 s, top micrograph; before). After prolonged (seconds) near-UV (405 nm) illumination over a large field of view (objective 20x, NA = 1, digital zoom 0.7) using maximal laser power (see section “Materials and Methods” for details on laser power), we observed stable increases in fluorescence over a very broad range (mean photoactivation*;*Δ*F/F* = 6.88 ± 0.25), likely due to very different expression levels in different HEK293 cells, rather than resting Ca^2+^-levels (Figure 2B, 80–120 s and bottom micrograph; after). This clone also showed that, whereas the 561 nm-induced emission spectrum of R-GECO1_H_ prior photoactivation was indistinguishable from that of the parent R-GECO1*wt* (Figure 2C, red plot), the spectrum obtained by 405 nm illumination was strikingly different, namely, retained blue emission but with a complete loss of the second peak of red fluorescence (Figure 2C, blue plot and arrowhead). We then expressed the clone in HeLa cells with the intention to monitor activity as these cells are more Ca^2+^-active. Transfected HeLa cells exhibited lower basal fluorescence than levels in HEK293T cells, albeit detectable (Figure 2D, top micrograph). Of note, low, but extant, fluorescence allowed us to calculateΔ*F/F* values without introducing near-zero division artifacts (see section “Materials and Methods” for more details). Importantly, we deem visible basal fluorescence as a highly useful feature for detecting cells that express the clone prior photoactivation. This eliminates the need to introduce additional non-PA optical markers. In fact, basal fluorescence is so low in PA-GFP, and also in PA-GCaMP, therefore requiring imaging with low intensity 405 nm (that does not cause substantial photoactivation) for spotting positive cells; to then target for photoactivation (e.g., see protocol in Berlin et al., 2015). Owing to low levels of fluorescence, we performed gradual photoactivation to minimize photobleaching, consisting of shorter and repeated bouts of 405 nm until reaching maximal photoactivation (seen as steady state fluorescence). In HeLa cells, this protocol demonstrated that R-GECO1_H_ underwent weak photoactivation (Figure 2D and Table 1). It has also revealed, unexpectedly, that this clone no longer exhibited the light-induced artifacts as seen with R-GECO1*wt* (compare Figure 1 to Figure 2D, arrowheads). These observations rule-out the hypothesis that the light-induced artifacts directly stem from instantaneous maturation of the blue-emitting population. Importantly, this clone exhibited very robust Ca^+2^-performance in response to varying challenges commonly employed; histamine or excessive extracellular Ca^2+^ (Figure 2E, left and right panels, respectively). Thus, the low extent of photoactivation was complemented by very large Ca^2+^-responses. We then expressed the clone in rat hippocampal neurons where R-GECO1_H_ exhibited similar characteristics to those obtained from HeLa cells, namely, very low basal fluorescence and weak photoactivation (Figure 2F). As our primary goal is to express these probes in neurons, the quantitative similarities in basal fluorescence (likely due to similar expression levels) and the degree of photoactivation in HeLa and primary neurons indicated to us that HeLa cells are better suited for predicting expression/performances of our clones in neurons instead of HEK293 cells.

**TABLE 1:** Summary of basal expression, extent of photoactivation, and Ca^2+^-performance.

HeLa cells	Mutation	Expression via 561 nm illumination (also basal fluorescence prior photoactivation)	Artifacts	Photoactivation	Ca^2+^ response
R-GECO1 (mApple)	None (WT)	+++	+++	Transient	3.48 ±0.76, *n* = 19
	I78T PA-R-GECO1	+++	−	2.5 ± 0.24, *n* = 16 (HEK: 2.8 ±0.23, *n* = 13) (neurons: 1.5 ± 0.18, *n* = 13)	1.31 ± 0.37, *n* = 10 (3.04 ± 0.18, *n* = 45) (neurons: 4.27 ± 0.77, n = 16)
	I78H	+++	−	0.61 ± 0.04, *n* = 6 (HEK: 6.88 ± 0.25, *n* = 125)	2.06 ± 1.15, *n* = 9
	I78A	++	−	0.96 ± 0.17, *n* = 2	1.79 ± 0.63, *n* = 5
	I78V	++	+++	0.82 ± 0.12, *n* = 4	1.44 ± 0.44, *n* = 6
	I78Y	+	++	1.94 ± 0.15, *n* = 6	-
	I78S	+	−	1.76 ± 0.114, *n* = 3	-
	I78E	++	−	1.33 ± 0.18, *n* = 11	-
	I78Q	+	−		-
	I78L	+++	++	Transient	0.84 ± 0.52, *n* = 2
	I78K	−			
	I78G	−			
	I78F	−			
	I78D	−			
	I78C	−			
	I78P	−			
	I78R	−			
	I78N	−			
	I78W	−			
R-GECO1.2 (mPlum)	Non (WT)	+++	+++	Transient	8.22 ± 1.03, *n* = 37
	I78L	+++	−	1.31 ± 0.09, *n* = 36	15.39 ± 1.65, *n* = 67
	I78T PA-R-GECO1.2	+++	−	1.2 ± 0.13, *n* = 31 (in HEK: 4.64 ± 0.34, *n* = 22) (neurons: 2.3 ± 0.22, *n* = 5)	23.80 ± 2.83, *n* = 49 (in HEK: 3.18 ± 0.29, *n* = 11)
	I78N	++	−	0.4 ± 0.17, *n* = 6	-
	I78H	++	Inverse	0.9 ± 0.26, *n* = 6	-
	I78A	++	−		-
	I78Y	+	−		-
	I78Q	+	−	0.5 ± 0.03, *n* = 8	-
	I78PRO	+	−		-
	I78C	+	−		-
	I78K	+	−		-
	I78G	+	−		-
	I78F	−			
	I78D	−			
	I78E	−			
	I78S	−			
	I78R	−			
	I78val	−			
	I78W	−			
jR-GECO1a (mApple)	I131T	+++	−	2.12 ± 0.129, *n* = 19 (in HEK: 14.28 ± 0.84, *n* = 53)	1.9 ± 0.18, *n* = 3
	I131H	−	−		
	I131R	−	−		
RCaMP1h (mRuby)	None (WT)	+++	+++	Transient	Not tested
	H115T	+++	+++	in HEK: 45.38 ± 2.03, *n* = 96	0.8 ± 0.53, *n* = 96
jRCaMP1A (mRuby)	H134I	+++	−	7.44 ± 3.74, *n* = 15 (in HEK: 12.07 ± 1.17, *n* = 33)	1.01 ± 0.10, *n* = 10 (in HEK: 1.22, n = 1)
	H134T	+++	−	4.83 ± 0.18, *n* = 25	0.33 ± 0.11, *n* = 3
jRCaMP1B (mRuby)	H134I	+++	−	6.9 ± 1.9, *n* = 21	0.53 ± 0.09, *n* = 8
	H134T	+++	−	2.93 ± 0.55, *n* = 23	0.28 ± 0.07, *n* = 7
	H134K	−	−		
	H134R	−	−		
mRuby3	H200I	+++	−	13.63 ± 1.37, *n* = 29	−
	H200T	+++	−	4.9 ± 0.29, *n* = 23	−
					

Dissatisfied with the PA performance of R-GECO1_H_, in all cell types, we were motivated to pursue our screen for better R-GECO mutants specifically in HeLa cells. We proceeded to make 17 more substitutions (see primers list), as we could not determine *a priori* which mutation would endow the best performances in photoactivation and in Ca^2+^-responsivity. Though most of the variants did not express at all (e.g., Supplementary Figure 2a, R-GECO1-I78R), it was surprising to find that multiple mutations could confer photoactivatability, for instance, I78Y, -S, and -E (Supplementary Figure 2b and Table 1). It was also surprising that the leucine-mutant (R-GECO1*-*I78L), despite the residue’s high degree of homology to isoleucine as is originally found in R-GECO1*wt*, lost its direct 405 nm-induced red emission (Supplementary Figure 3a) and the large light-induced artifacts (Supplementary Figure 3b). Moreover, it did not display photoactivation. Unfortunately, this collection of variants (including R-GECO1*-*I78L) failed to respond to Ca^2+^ (e.g., Supplementary Figure 3c for R-GECO1-I78L) and were not further characterized (see summary in Table 1).

**FIGURE 3:**
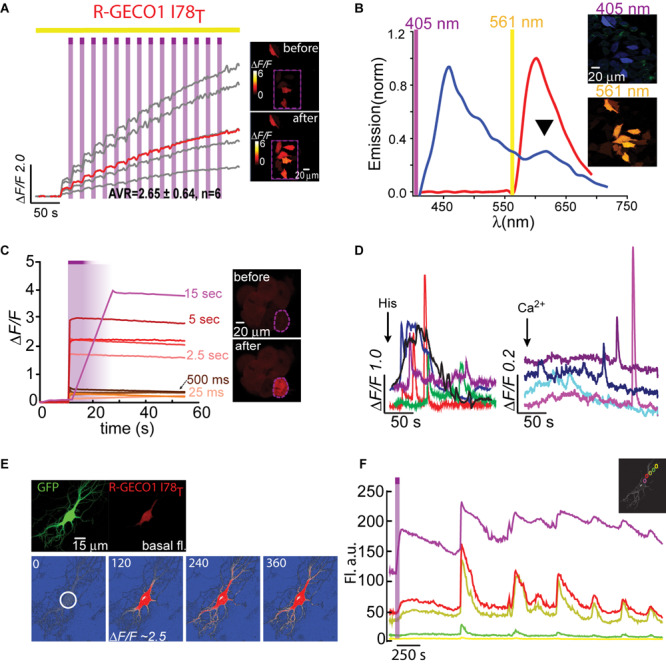
Photoactivatable R-GECO1_T_. **(A)** Stable photoactivation of R-GECO1_T_ in HeLa cells. Cells imaged by 561 nm (yellow bar) are intermittently excited by short (1 s) bouts of 405 nm (magenta bars) exhibit stable increase in Ca^2+^-independent fluorescence, until reaching a plateau. Insets—HeLa cells imaged by 561 nm before (top) and after (bottom) photoactivation (magenta dashed square). **(B)** Emission spectra of R-GECO1_T_ collected from HeLa cells a before photoactivation by use of 405 nm (blue plot and top micrograph) or 561 nm (red plot and bottom micrograph). Note the diminished 405 nm-induced red emission (black arrowhead). **(C)** R-GECO1_T_ exhibits rapid photoactivation. Very brief photoactivation bouts (25–500 ms) yield robust photoactivation. 5 s 405 nm illumination yields 85% of that obtained by longer exposures (15 s). Insets—single cell before (top) and after (bottom) photoactivation (magenta dashed region). **(D)** R-GECO1_T_ is functional as a Ca^2+^-probe. Representative traces showing Ca^2+^-activity following the application of histamine (15 μM, left) or Ca^2+^ (+50 μM, right). **(E)** Hippocampal neuron co-transfected with GFP and R-GECO1_T_, showing the noticeable basal fluorescence of this clone prior photoactivation (top right micrograph). Bottom images show spread of fluorescence, following somatic photoactivation of R-GECO1_T_ (white circle). **(F)** Ca^2+^-activity is larger in highlighted regions. Traces show that soma and nearby dendrites exhibit larger Δ*F/F* than distant dendrites.

We lastly engineered R-GECO1_T_ (R-GECO1-I78T) and expressed it in HeLa cells. Notably, we did not expect this clone to display any photoactivation based on observations with PA-GFP in which T203H enables photoactivation, whereas H203T cancels it. Counterintuitively, R-GECO1_T_ exhibited the largest extent of photoactivation of all clones tested, thus far, in HeLa cells (Δ*F/F* = 2.65 ± 0.64, *n* = 6) by short bouts of 405 nm illumination (Figure 3A). Akin to R-GECO1_H_, this mutation completely eliminated the light-dependent artifacts (Figure 3A), without abolishing the blue-emitting population (Figure 3B, blue plot). However, this mutation significantly reduced the appearance of the direct excitation of the red-fluorophore by 405 nm in comparison to R-GECO1*wt* (Figure 3B, arrowhead). We also noticed that single photoactivation bouts yielded very rapid and sharp increases in fluorescence, suggesting to us higher sensitivity to photoactivation. We tested different photoactivation protocols on single cells and found that efficient photoactivation could be obtained very rapidly by irradiating the cells with as brief as 500 ms bouts of 405 nm (Figure 3C). Lastly, R-GECO1_T_ retained its responsiveness to Ca^2+^ (Figure 3D), which increased after photoactivation (Figure 3F and Supplementary Figure 4a). These responses were on par with the responses obtained for R-GECO1_H_ as well as for R-GECO1*wt* under similar conditions (compare Figures 1, 2, summarized in Table 1). In cultured hippocampal neurons, R-GECO1_T_ exhibits detectable basal fluorescence (Figure 3E, top micrograph and Figure 3F, top magenta plot starts with noticeable fluorescence), undergoes photoactivation and readily reports on action potential (AP) firing (Figures 3E,F). Together, we find that R-GECO1_T_ displayed the best combination of features, namely, moderate extent of photoactivation, large Ca^2+^-responsiveness, reduced artifacts, and reduced direct excitation of the mature red chromophore. We therefore denoted this clone PA-R-GECO1 (Table 1).

We were then curious as to whether the photoactivation of R-GECO1_T_ showed dependence on Ca^2+^ ions (as observed for another photoconvertible GECI denoted CaMPARI; Fosque et al., 2015). To address this, we expressed R-GECO1_T_ in HEK293 cells for these are typically less Ca^2+^-active than HeLa cells (Morita et al., 2015), thereby with likely lower cytoplasmic Ca^2+^ levels. We photoactivated cells in regular imaging medium (as used in experiments with HeLa cells), as well as in media with or without Ca^2+^ (Supplementary Figure 4b and see section “Materials and Methods”). First, as seen with other clones, HEK293 cells showed much more robust (and variable) expression of R-GECO1_T_ than when expressed in HeLa cells or in neurons (under similar conditions; “standard medium”). This translated into much higher degrees of photoactivation. More specifically, both standard- and Ca^2+^-free media enabled cells to undergo ∼8 (Δ*F/F* = 7.08 ± 0.11, *n* = 263) and ∼7 fold (Δ*F/F* = 6.23 ± 0.20, *n* = 181) increases in fluorescence (Supplementary Figures 4b,c, red and gray plots, respectively; raw data in Supplementary Figures 4d,e), whereas the presence of Ca^2+^ significantly reduced the ability of the clone to undergo photoactivation (Δ*F/F* = 0.71 ± 0.01, *n* = 294), even to lower extents than seen in HeLa cells (Supplementary Figure 4c, blue plot and Supplementary Figure 4f; compare with Figure 3). These results show that the photoactivation of R-GECO_T_ does not depend on Ca^2+^, rather it diminishes it. We also find no changes in the fluorescence emission, before and after photoactivation, (regardless the media) when illuminated with 561 nm (Supplementary Figure 4g). However, the emissions resulting from 405 nm illumination did change after photoactivation—the direct red emission (at ∼600 nm) was strongly increased by 405 nm irradiation no matter the medium (Supplementary Figure 4h). These observations show that the chromophore undergoes irreversible chemistry by 405 nm (as is the case for PA-GFP and that this modification gives rise to enhanced direct absorption of 405 nm. Since this clone does not exhibit artifactual behavior as the original R-GECO1, it strengthens our hypothesis that the artifacts do not stem from direct red emission by 405 nm illumination, rather a transient change in the chromophore induced by 405 nm, most likely isomerization, and the I to T mutation disrupts or abolishes this ability while increasing 405 nm absorbance.

### Next-Generation PA-R-GECI

**FIGURE 4:**
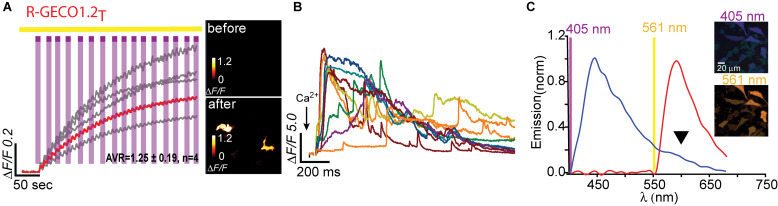
Photoactivatable R-GECO1.2_T_. **(A)** Stable photoactivation of R-GECO1.2_T_ in HeLa cells. Cells imaged by 561 nm (yellow bar) are intermittently excited by short (1 s) bouts of 405 nm (magenta bars) exhibit stable increase in Ca^2+^-independent fluorescence, until reaching a plateau. Insets—HeLa cells imaged by 561 nm before (top) and after (bottom) photoactivation (magenta dashed square). **(B)** R-GECO1.2_T_ is functional as a Ca^2+^-probe. Representative traces showing Ca^2+^-activity following the application of Ca^2+^ (+50 μM). **(C)** Emission spectra of R-GECO1.2_T_ collected from HeLa cells before photoactivation by use of 405 nm (blue plot and top micrograph) or 561 nm (red plot and bottom micrograph). Note the diminished 405 nm-induced red emission (black arrowhead).

Having seen that a single point mutation could confer most of the traits desired in a PA Ca^2+^-probe, we were curious as to whether we could quickly engineer other PA-R-GECI with higher contrast using the same strategy. Naturally, we moved to an optimized version of R-GECO1 probe, namely, R-GECO1.2 (Wu et al., 2013). Of note, R-GECO1 and -1.2 are not based on the same RFP backbone, rather R-GECO1.2 is based on mPlum. However, mApple and mPlum both share the I78 residue (Supplementary Figure 1). We then generated 18 different R-GECO1.2 clones and found that most mutants failed to express, as seen with their R-GECO1 counterparts (Table 1), but with a few exceptions. For instance, whereas R-GECO1_L_ almost lost its ability to sense Ca^2+^, R-GECO1.2_L_ remained potent (compare Supplementary Figures 3a,b). The best performing clone was R-GECO1.2_T_, displaying moderate photoactivation (Δ*F/F* = 1.25 ± 0.19) in HeLa cells (though to lower extent than R-GECO1_T_), but with significantly larger Ca^2+^-responses (Δ*F*/*F* = 23.80 ± 2.83) (Figures 4A,B and Table 1). It also displayed strongly diminished red emission by direct 405 nm irradiation, not to mention loss of the near-UV light-induced artifacts (Figures 4A,C arrowhead). We also find that, analogous to R-GECO1_T_, this variant does not exhibit Ca^2+^-dependent photoactivation (Supplementary Figures 5a,b) and 561-nm induced emission remain similar before and after photoactivation, with or without Ca^2+^ (Supplementary Figure 5c). Its emission in response to 405 nm, before and after photoactivation, showed the same trend as R-GECO1_T_, but to larger extents. More specifically, photoactivation enhanced the direct red emission by 405 nm (Supplementary Figure 5d). Moreover, photoactivation also strengthened the emission of another peak, ∼512 nm (Supplementary Figure 5d, but also see Supplementary Figure 4d). This supports the notion that 405 nm irradiation causes a chemical change in the vicinity of the chromophore (likely by β-elimination; Miyawaki et al., 2012), and therefore creates a new stable chromophore with increased green emissions. We therefore denoted this clone PA-R-GECO1.2.

**FIGURE 5:**
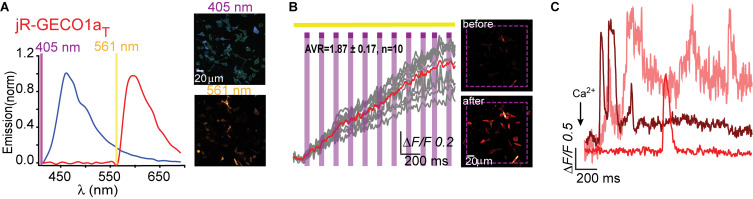
Photoactivatable jR-GECO1a_T_. **(A)** Emission spectra of jR-GECO1a_T_ collected from HeLa cells before photoactivation by use of 405 nm (blue plot and top micrograph) or 561 nm (red plot and bottom micrograph). **(B)** Stable photoactivation of jR-GECO1a_T_ in HeLa cells. Cells imaged by 561 nm (yellow bar) are intermittently excited by short (1 s) bouts of 405 nm (magenta bars) exhibit stable increase in Ca^2+^-independent fluorescence. Insets—HeLa cells imaged by 561 nm before (top) and after (bottom) photoactivation (magenta dashed square). **(C)** jR-GECOa_T_ is functional as a Ca^2+^-probe. Representative traces showing Ca^2+^-activity following the application of Ca^2+^ (+50 μM).

We then turned to test the newest R-GECO variant, jR-GECO1a; also based on mApple (Dana et al., 2016). Based on our experience with R-GECO1 and -1.2, we only produced three variants (I131T, H, and R), expecting these to provide the entire range of features, namely, T and H to perform well and R to serve as control for a poor expression variant. Indeed, jR-GECO1a-I131T behaved as PA-R-GECOs, explicitly retained blue population, lacked direct 405 nm-induced red emission and artifacts and exhibited brightest features (photoactivation, Δ*F/F* = 2.12 ± 0.13; Ca^2+^-performance, Δ*F/F* = 1.9 ± 0.13) (Figures 5A–C). Nevertheless, these were of lower amplitudes than those of PA-R-GECO1 and -1.2. In parallel, we explored a closely related R-GECI family denoted RCaMPs (RCaMP1h; Akerboom et al., 2013 and jRCaMP1a, -b; Dana et al., 2016; see Dana et al., 2016; Kerruth et al., 2019 for GECIs genealogy). RCaMPs are based on mRuby and therefore enclose a histidine in the 203rd position (H115, RCaMP1h numbering) (Supplementary Figure 1). We briefly examined the basic properties of the three RCaMPs using our protocols and found that, akin to R-GECOs, 405 nm excitation yielded blue emitting populations, but without direct excitation of the red chromophore (Supplementary Figure 6a). RCaMP1h also displayed similar light-induced artifacts as observed for R-GECO1 and -1.2 (Supplementary Figure 6b), whereas jRCaMPa did not (Supplementary Figure 6c).

**FIGURE 6:**
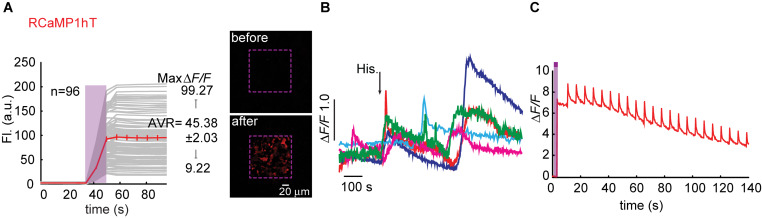
Photoactivatable RCaMP1h_T_. **(A)** Stable photoactivation of RCaMP1h_T_ in HEK293 cells. RCaMP1h_T_ in HEK293T cells during prolonged photoactivation with 100% of 405 nm at ∼1.7 mW (magenta bar). Insets—HEK cells imaged by 561 nm before (top) and after (bottom) photoactivation (magenta dashed square). **(B)** RCaMP1h_T_ is a poor Ca^2+^-probe. Representative traces showing Ca^2+^-activity following the application of histamine (15 μM). **(C)** RCaMP1h_T_ maintains its artifacts. Following photoactivation, brief illumination by 405 nm yields large Ca^2+^-independent increases in fluorescence.

We began engineering H115T mutants because, so far, substitution to a threonine yielded best performances. We also made a H115I substitution because we were curious to see the potential involvement of this in the transient photoactivation seen in all R-GECOs, including RCaMP1h. Surprisingly, all six clones exhibited robust and stable photoactivation; much larger than most of the responses obtained for the different R-GECO variants (Supplementary Figure 7). Notably, RCaMP1h_T_ exhibited the largest extent of photoactivation with contrast as high as ∼100 fold in HEK293 cells (mean photoactivation, Δ*F/F* = 45.38 ± 2.03, *n* = 96) (Figure 6A). However, all RCaMP-variants did not yield very strong Ca^2+^-responses (Figure 6B and Supplementary Figure 7). In general, we note a weak, albeit non-significant, inverse relationship between Ca^2+^-performance and extent of photoactivation among all R-GECO and RCaMP variants tested (Supplementary Figure 7g). We hypothesize this to stem from the fact that both capabilities compete for the same mechanism, namely, deprotonation of the chromophore and increase in fluorescence. In other words, the addition of Ca^2+^ (akin to increase in basal fluorescence) reduces the effective range for photoactivation, while maintaining the same total fluorescence (see Supplementary Figures 4d–f). Lastly, RCamP1h_T_ retained its light-induced artifacts by 405 nm irradiation (Figure 6C). Thus, owing to these limitations, we did not continue with the RCaMP family for further development of red PA Ca^2+^-probes, but we did find their FP backbone to be highly relevant for the generation of novel PA-RFPs (below).

### Engineering a Novel PA-mRuby3

**FIGURE 7:**
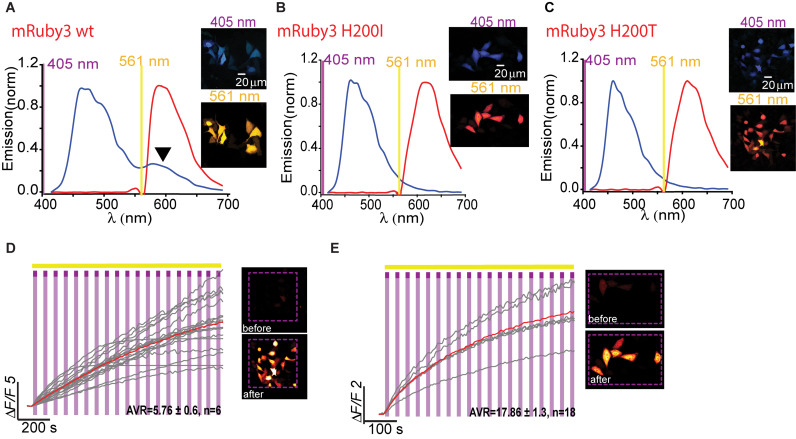
Photoactivatable mRuby3. Emission spectra of mRuby3-wt **(A)**, mRuby3 H200I **(B)**, and mRuby3 H200T **(C)** collected from HeLa cells before photoactivation by use of 405 nm (blue plots and top micrographs) or 561 nm (red plots and bottom micrographs). Note the 405 nm-induced red emission in mRuby3-wt (**A**, black arrowhead), but complete loss in mRuby3 H200I, and H200T (**B,C**, respectively). Robust photoactivation of mRuby3 H200I **(D)** and mRuby3 H200T **(E)** in HeLa cells. Cells imaged by 561 nm (yellow bar) are intermittently excited by short (1 s) bouts of 405 nm (magenta bars) exhibit stable increase in Ca^2+^-independent fluorescence. Insets—HeLa cells imaged by 561 nm before (top) and after (bottom) photoactivation (magenta dashed square).

Owing to the fact that RCaMPs, in particular RCaMP1-H115I and H115T, showed very robust photoactivation among the clones tested, we wondered whether it would be possible to use this knowledge for rational engineering of a novel PA-RFP, namely, PA-mRuby. Notably, there is no PA-mRuby. We therefore turned to the newest and brightest mRuby denoted mRuby3 (Bajar et al., 2016). We mutated its corresponding 203rd residue (i.e., H200) to either isoleucine or threonine; producing mRuby3-H200I and -H200T. Analysis of emission spectra showed that both variants did not show any 405 nm-induced emissions by the chromophore as compared to their parent protein with its minimal, albeit noticeable, direct excitation (Figure 7A, arrowhead, Figures 7B,C). Importantly, both variants rendered mRuby3 photoactivatable, with mRuby3-H200I exhibiting more potent photoactivation (Δ*F/F* = 13.63 ± 1.37, *n* = 29) in HeLa cells (Figures 7D,E). Therefore, we denoted this clone PA-mRuby3_I_.

## Discussion

Here, we describe a simple rational design strategy with which we have created a palette of unique PA red-GECIs, along a novel PA-RFP. These, unlike PA-GFP and PA-RFPs, only required a single point mutation (equivalent to T203H mutation as in PA-GFP; Baird et al., 1999; Patterson and Lippincott-Schwartz, 2002). Moreover, these variants did not need to be optimized for better folding and emission at physiological conditions (pH and temperature), as was the case with green PA-GECI (Berlin et al., 2015) and PA-GEVI (Lee et al., 2019). In a previous attempt to create a PA version of R-GECO1 (Hoi et al., 2012), the authors introduced multiple mutations as are found in PA-mCherry. However, since R-GECO1 is not based on mCherry, this scheme likely yielded low photocontrast and, more importantly, this clone displayed poor Ca^2+^ responses.

Substitution of isoleucine to threonine at position 78 (I78T) in mApple or mPlum-based probes, namely, R-GECO1 and -1.2, respectively, resulted in generating functional and PA probes. More specifically, PA-R-GECO1 and -1.2 express efficiently at 37°C in HEK293, HeLa cells, as well as in cultured primary neurons, undergo robust 405 nm-induced photoactivation and readily report on Ca^2+^-activity (especially PA-R-GECO1.2 with Ca^2+^-dependent increases in fluorescence of up to 24 fold). In addition, these display low to moderate basal florescence (e.g., Figure 2B, pre-photoactivation), a trait we deem highly useful for initial detection of cells that express the clone prior photoactivation, without the need of additional fluorescent markers (such as GFP) or illumination with low-intensity near-UV (Berlin et al., 2015). Importantly, these probes also show greatly diminished (or completely eliminated) artifactual behavior which should make them ideal for concomitant use with many blue light-absorbing optogenetic tools, such as blue-absorbing opsins (Akerboom et al., 2013; Wu et al., 2013). We also report on the rapid engineering of a high-contrast PA-mRuby3_I_. Notably, PA-mRuby3_I_ is based on a highly optimized, bright, and stable monomeric RFP (Piatkevich and Verkhusha, 2010).

Although beyond the scope of this work, our systematic comparison between different mutations within multiple GECIs also sheds light on the transient photoconversion mechanism of R-GECOs. We observe that most variants can no longer emit red fluorescence when illuminated by near-UV light, despite retaining a blue-emitting population (Figures 3, 4). This observation rules-out the involvement of direct 405 nm-excitation of the red chromophore as the culprit for the transient increases in fluorescence. It also rules-out the instantaneous light-dependent maturation of the blue chromophore. The fact that many different mutations introduced around the chromophore abolished photoconversion and stabilized photoactivation support the notion that, unlike in GFP, light induces a chemical shift in the chromophore that is sufficiently long-lived (hundreds of ms to seconds) to enable increase in absorbance of 561 nm, so that when we revert back to 561 nm imaging of the probe, this is seen as a large increase in Ca^2+^-independent fluorescence that disappears within half a second. Such long-lived isomerization of the red chromophore to an anionic fluorescent state (Wu et al., 2013), could then allow for, akin to GFP, further permanent chemical changes such as decarboxylation of the E222 residue (GFP numbering, Patterson and Lippincott-Schwartz, 2002; van Thor et al., 2002).

We note that probes that exhibit better photoactivation are also probes that tend to respond less well to Ca^2+^. For GCaMPs, binding of Ca^2+^-ions changes their absorption spectrum, effectively increasing their ability to absorb 488 nm (i.e., the imaging wavelength) (Barnett et al., 2017). However, in the case of R-GECIs, binding of Ca^2+^-ions can also increase their quantum yield (Molina et al., 2019). In fact, R-GECO and RCaMP exhibit very different mechanisms that engender increase in fluorescence when bound to Ca^2+^. Specifically, whereas the fluorescence change in R-GECO is mostly due to a dramatic increase in the excitable protonation state (i.e., shift in absorption spectrum), akin to GCaMPs, RCaMPs (RCaMP1h and jRCaMPa or b) belong to a second class in which the increase in fluorescence depends on both Ca^2+^-induced shift in fluorescence and in the quantum yield of the excitable state. Thus, in the case of R-GECI, it can be assumed that mutations around the chromophore that enhance photoactivation limit the subsequent increase in absorption following Ca^2+^-binding. Conversely, studies of R-GECO’s unique photoconversion behavior show that short wavelengths (405 and 488 nm) shift the absorbance of the calcium-bound population of R-GECO1 to the right therefore decreasing its fluorescence (by up to 40%) when illuminated with its optimal imaging wavelength (i.e., 561 nm) (Akerboom et al., 2013). Thus, it can be assumed that after loading the probe with calcium, its absorption of short wavelengths (employed for photoactivation) is also decreased and therefore would yield lower photoactivation. Inversely, when devoid of Ca^2+^, illumination of R-GECO1 by short wavelengths results in an apparent decrease in the absorbance of protonated chromophore and a simultaneous increase in absorbance in deprotonated chromophore. In this case, it can therefore be assumed that stable photoactivation would mimic this scenario, namely, enabling R-GECO to absorb 561 better when it is devoid of Ca^2+^, thereby performing less well when saturated by Ca^2+^ (Akerboom et al., 2013; Molina et al., 2019).

Lastly, we could not find any correlation between the property of the amino acids (hydrophobicity, polarity, or volume) and the extent of photoactivation or Ca^2+^-performance (Table 2). This is not completely surprising as estimating how an amino acid substitution would affects the entire network around the chromophore, *a priori*, is extremely challenging without detailed spectroscopic analysis (Heim et al., 1994). Future spectroscopic work should shed light on the nature of these phenomena.

**TABLE 2:** Lack of correlation between amino acid properties and extent of photoactivation and Ca^2+^-performance.

Amino acid	Polarity^1^	Hydrophobicyty^2^	Volume^3^	Extent of photoactivation of R-GECO1	Ca^2+^-response
Ser	9.2	0.76	60.8	1.76	1.31
Arg	10.5	1.03	111.5		
Leu	4.9	–21.91	107.5		
Pro	8	–0.49	81		
Thr	8.6	0.59	77.1	2.5	
Ala	8.1	0.96	60.6	0.96	1.79
Val	5.9	–3.74	91.3	0.82	1.44
Gly	9	1.12	43.5		
Ile	5.2	–15.16	107.5		
Phe	5.2	–17.28	121.3		
Tyr	6.2	–15.09	123.6	1.94	
Cys	5.5	0.69	72.5		
His	10.4	1.03	99.3	0.61	2.06
Gln	10.5	1.21	93.9		
Asn	11.6	1.17	78		
Lys	11.3	0.92	108.5		
Asp	13	0.96	74.1		
Glu	12.3	0.86	90.4	1.33	
Met	5.7	–3.01	105.1		
Trp	5.4	–9.63	144.1		

*^1^Grantham, R. Amino Acid Difference Formula to Help Explain Protein Evolution. Science 185, 862-864 (1974). ^2^Bull, H.B. & Breese, K. Surface tension of amino acid solutions: a hydrophobicity scale of the amino acid residues. Arch Biochem Biophys 161, 665-670 (1974). 3. Kyte, J. & ^3^Zamyatnin, A.A. Protein volume in solution. Progress in Biophysics and Molecular Biology 24, 107–123 (1972).*

Together, we describe a fast and simple method for generating PA-GECIs from the R-GECO and RCaMP family. The best performing probes, namely, R-GECO1_T_ and -1.2_T_, display basal fluorescence that can be imaged by very low 561 nm laser powers. This should therefore allow to detect the probes *in vivo* by use of two-photon imaging (Drobizhev et al., 2014). In addition, photoactivation by 405 light can be obtained via wide-field epifluoresence, as previously shown for similar probes at similar laser powers (e.g., CaMPARI; Ebner et al., 2019) or via light-guides (Sileo et al., 2015). However, as short wavelengths poorly penetrate tissues and scatter (Carmi et al., 2019), it would seem more attractive should these probes be compatible with two-photon illumination. Though we do not test for the latter in this work, previous work show the feasibility of photoconversion and imaging of RFPs (mApple and mPlum in particular) using multiphoton lasers (Drobizhev et al., 2009, 2014; Molina et al., 2019).

## Conclusion

The ability to generate numerous PA-GECIs, by incorporation of a single point mutation at position 203 (GFP numbering), suggests that this mechanism is not restricted to a handful of RFP and should therefore be transferrable to other R-GECIs (and likely other RFPs). We anticipate that the PA-GECIs developed here will serve as a starting point for the development of additional or enhanced versions (of larger photoactivation contrast and larger Ca^2+^-responses) by additional methods, such as directed evolution, and expect to see the emergence of new PA-R-GECIs in the very near future.

## Materials and Methods

### cDNA Constructs and Site Directed Mutagenesis

R-GECO1, R-GECO1.2, RCaMP1h, and jRCaMP1a/b were purchased from Addgene (addgene clones: # 32444, 45494, #32444, #42874, respectively). Point mutations were introduced by PCR, saturating position I78 (equivalent to T203 in GFP) by all other amino acids: I78L, I78T, I78N, I78H, I78A, I78Y, I78Q, I78P, I78C, I78K, I78G, I78F, I78D, I78E, I78S, I78R, I78V, I78W (see list of primers at Supplementary Table 1). PCR reactions were carried out with the use of Pfu polymerase (Promega, United States), at annealing temperatures of 60°C (1:30 min); extension at 68 °C (15 min). RCaMP1h was removed from its original bacterial expression vector using standard PCR reaction and inserted into a mammalian expression vector.

### Tissue Culture

Heterologous cell lines, HEK293 or HeLa cells, were grown to ∼50% confluence on poly-D-lysine-coated 12 mm coverslips (Bar-Naor, Israel) and transfected with 0.5–1 μg plasmid DNA with the use of ViaFect (Promega, United States) for overnight incubation in Dulbecco‘s modified Eagle’s medium (DMEM) with 5–10% fetal bovine serum (FBS) at 37°C and 5% CO_2_. Primary neuronal hippocampal neurons were collected as previously described (Berlin and Isacoff, 2018). Briefly, hippocampi were harvested from rat neonates (P0-1) and plated at a density of ∼100K cells/well (24 well-plate) on PDL-coated 12 mm glass coverslips, grown at 37°C. At 7 days *in vitro* (DIV), neurons were transfected with 0.5–1 μg DNA of the different variants by the calcium-phosphate transfection method (Berlin and Isacoff, 2018) and imaged at 9–13 DIV. All animal procedures were approved by the Technion’s Institutional Animal Care and Use Committee (permit no. IL-130-09-17).

### Imaging

Imaging was performed on a Zeiss Laser Scanning Confocal Microscope equipped with a with a spectrally resolved 32-pixel GaAsP detector array (LSM-880-meta detector; Zeiss, Germany). We monitored emission at 8 nm intervals. Excitation of R-GECIs was performed by 405 and 561 nm lasers. Brief pulses of 405 nm (typically rastering over defined regions of interest at 0.64 μs/pixel dwell time). The output power of the 405 nm laser line was ∼3.4 mW when using an air objective (10x/0.45). It is measured at 100% power slider without laser blinking (i.e., bi-directional scanning). Our experiments were always performed by uni-directional scanning, hence the power is corrected by a factor of ∼0.5, hence ∼ 1.7 mW. Our experiments were conducted using a water immersion objective lens 20x [a water Plan-Apochromat objective lens; 20x/1.0 DIC D = 0.17 (UV) VIS-IR M27 75 mm] with a focal spot diameter of 0.5 μm (D = 1.2 ^∗^ λ/NA). This lens transmits identically to the one used for power measurements of the 405 nm laser line and it illuminates an area on the sample of 0.2 μm^2^. The power density (PD) under these conditions is ∼10^6^ W/cm^2^. The AOTF transmission is calibrated to be linear so that 1% laser power slider is equivalent to 10 KW/cm^2^ @ 405 nm. Cells were imaged in a standard imaging solution containing (in mM): 138 NaCl, 1.5 KCl, 1.2 MgCl_2_, 2.5 CaCl_2_, 10 D-glucose, 10 HEPES, pH 7.4. Ca^2+^-imaging was performed for several minutes with low intensity 561 nm to avoid bleach. To test functionality, we applied histamine (15 μM; final concentration) or additional Ca^2+^ (denoted Ca^2+^-challenge, +50 μM) when using HeLa or HEK293 cells, as previously described (Berlin et al., 2015). For imaging photoactivation in Ca^2+^-Free or Ca^2+^-saturated states, cells were either incubated for 10 min in nominally Ca^2+^-free imaging medium supplemented with the Ca^2+^-chelator EDTA (5 mM) and a Ca^2+^-ionophore; ionomycine (10 μM). This treatment induces Ca^2+^-release and promotes Ca^2+^-depletion from cells. For saturating Ca^2+^, we incubated cells with extracellular Ca^2+^ (2 mM) and ionomycine (10 μM) (McCombs and Palmer, 2008). Image acquisition and photoactivation (i.e., bleach mode; Zen software, Zeiss) are performed intermittently so that no image (i.e., fluorescence) is acquired during illumination with 405 nm. Time resolved fluorescence plots thereby present data from images acquired before and after the 405 nm photoactivation bouts. Construction of a continuous fluorescence plot is done by connecting the data from images acquired before photoactivation to the images following the bout (appearing as slopes, e.g., Figure 3; 15 s).

### Statistical Analysis

Change in fluorescence (Δ*F/F*) was calculated by (*F*_t_–*F*_0_)/*F*_0_, where *F*_t_ is measured fluorescence (in arbitrary units, a.u.) at a given time *t* and *F*_0_ is initial baseline fluorescence, typically calculated from averaging the 10 first images. Δ*F/F* = 1 describes an increase by 100%, equivalent to twofold increase in fluorescence. Owing to easily observable basal fluorescence (see Supplementary Figures 4e–g for multiple examples), we did not need encounter near division-by-zero artifacts. For collecting emission spectra, we used small imaging intervals (8 nm, see above) which allowed us to plot the data as smooth (splined) curves rather than straight lines (SigmaPlot 11). We find no differences between the two plots (e.g., Supplementary Figure 5e). All results are displayed as mean ± SEM. Sample sizes are provided (summarized in Table 1). Significance was tested using one-way ANOVA (*post hoc* Tukey) where relevant and relationship between Ca^2+^-performance and extent of photoactivation was tested by Spearman correlation using SigmaPlot 11.

## Data Availability Statement

All datasets generated for this study are included in the article/Supplementary Material.

## Ethics Statement

The animal study was reviewed and approved by the Technion’s Institutional Animal Care and Use Committee (permit No. IL-130-09-17).

## Author Contributions

WH and SB designed, performed, and analyzed the experiments and wrote the manuscript.

## Conflict of Interest

The authors declare that the research was conducted in the absence of any commercial or financial relationships that could be construed as a potential conflict of interest.

## Supplementary Material

FIGURE S1 Sequence alignment of green and red fluorescent proteins. Homologous residues are color coded, whereas homology shared by all proteins is highlighted in yellow. Stars indicate the chromophore. Isoleucine residue in mPlum is indicated by blue circle. The T203 position (GFP numbering) is indicated by black arrowhead. Note that several PA-RFPs share positively charged amino acids (H or R) at the corresponding T203 position.

FIGURE S2 R-GECO1_R_ and R-GECO1_Y_. **(a)** R-GECO1_R_ does not express nor undergoes photoactivation in HeLa cells. Micrographs show HeLa cell before (left) and after (right) photoactivation (magenta dashed square). **(b)** R-GECO1_Y_ undergoes photoactivation in HeLa cells. Micrographs show HeLa cell before (left) and after (right) photoactivation (magenta dashed square).

FIGURE S3 R-GECO1_L_ retains light-induced artifacts. **(a)** Emission spectra of R-GECO1_L_ collected from HeLa cells before photoactivation by use of 405 nm (blue plot and top micrograph) or 561 nm (red plot and bottom micrograph). Arrowhead indicates the weak direct excitation of the red chromophore by 405 nm. **(b)** R-GECO1_L_ does not undergo photoactivation in HeLa cells. Cells imaged by 561 nm (yellow bar) are intermittently excited by short (1 s) bouts of 405 nm (magenta bars) does not exhibit stable increases in Ca^2+^-independent fluorescence but exhibits light-induced artifacts. **(c)** R-GECO1_L_ does not responding to Ca^2+^ (left), whereas R-GECO1.2_L_ does (right).

FIGURE S4 Characterization of the effect of Ca^2+^ on photoactivation and emission spectra of R-GECO1-I78T. **(a)** Ca^2+^-activity before and after photoactivation. Left: micrographs of R-GECO1-I78T expressed in HEK293 cells; initially (1), during photoactivation (2; before and after), and after photoactivation (3). Data are summarized in plot (right). Note the large increase in the amplitudes of the spontaneous Ca^2+^-oscillations after photoactivation (magenta bar). **(b,c)** Extent of photoactivation of R-GECO1-I78T under different conditions. **(b)** Micrographs of R-GECO1-I78T expressed in HEK293 cells in standard medium (top, red), Ca^2+^-free (middle, gray) and Ca^2+^-saturated (bottom, dark blue) conditions, before and after photoactivation. Data are summarized in **c**. Note that Ca^2+^-free and standard conditions are beneficial to photoactivation, whereas the presence of Ca^2+^ is detrimental. **(d–f)** Representative experiment showing raw fluorescence traces before and after photoactivation of HEK293 cells in different meda (as summarized in **c**). These show the detectable basal fluorescence (above zero) from which we could faithfully calculate Δ*F/F* (see section “Materials and Methods”). Traces also show that the baseline drastically changes, though maximal fluorescence is obtained under all conditions. Emission spectra induced by 561 nm illumination **(g)** or 405 nm **(h)**, under different conditions (color coded as in **e**).

FIGURE S5 Characterization of the effect of Ca^2+^ on photoactivation and emission spectra of R-GECO1.2-I78T. **(a)** Micrographs of R-GECO1.2-I78T expressed in HEK293 cells in standard medium (top, red), Ca^2+^-free (middle, gray), and Ca^2+^-saturated (bottom, dark blue) conditions, before and after photoactivation. **(b)** Extent of photoactivation of R-GECO1.2-I78T under different conditions (color coded as in **a**). Note that Ca^2+^-free and standard conditions are beneficial to photoactivation, whereas the presence of Ca^2+^ is detrimental. Emission spectra induced by 561 nm illumination **(c)** or 405 nm **(d)**, under different conditions (color coded as in **a**). Note the striking increase in the emission of 512 nm. **(e)** Comparison between straight and smoothed lines for plotting emission spectra. A representative emission spectrum induced by 561 nm. Data points are collected every 8 nm (open circles; see section “Materials and Methods”). Plot is constructed by straight lines (black) or smoothed (splined, red plot). Note the agreement between both plots.

FIGURE S6 Characterization of RCaMP1h and JRCaMP1b. **(a)** Emission spectra of RCaMP1h collected from HeLa cells before photoactivation by use of 405 nm (blue plot and top micrograph) or 561 nm (red plot and bottom micrograph). RCaMP1h **(b)** exhibits transient photoconversion during intervals of near UV light irradiation (405 nm), whereas jRCaMP1a **(c)** does not, rather undergoes slight photobleaching.

FIGURE S7 Photoactivatable JRCaMP1a and JRCaMP1b are poor Ca^2+^-probes. Emission spectra of JRCaMP1a_I_ **(a)** and JRCaMP1a_T_ **(b)** collected from HeLa cells before photoactivation by use of 405 nm (blue plots and top micrographs) or 561 nm (red plots and bottom micrographs). Stable photoactivation of JRCaMP1a_I_ **(c)**, JRCaMP1a_T_ **(d)**, JRCaMP1b_I_ **(e)**, or JRCaMP1b_T_ **(f)** in HeLa cells. Cells imaged by 561 nm (yellow bar) are intermittently excited by short (1 s) bouts of 405 nm (magenta bars) exhibit stable increase in Ca^2+^-independent fluorescence. Micrographs show HeLa cells imaged by 561 nm before (top) and after (bottom) photoactivation (magenta dashed square). (Right) Representative traces showing Ca^2+^-activity following the application of Ca^2+^ (+50 μM) of JRCaMP1a_I_ **(c)**, JRCaMP1a_T_ **(d)**, JRCaMP1b_I_ **(e)**, and JRCaMP1b_T_ **(f)**. **(g)** Correlation between extent of photoactivation and Ca^2+^-performance of various probes in HeLa cells and in HEK293 cells (inset). R-GECO1-I78T (red) and R-GECO1.2-I78T (blue) are highlighted. Note the inverse trend between the features (though non-significant, Spearman correlation, see section “Materials and Methods”).

TABLE S1 List of primers used to generate the various mutants tested. Mutated residues are highlited (yellow).
