# Supplementary material for: Red Photoactivatable Genetic Optical-Indicators
Source: Front Cell Neurosci. 2020 May 28;14:113. doi: 10.3389/fncel.2020.00113 (PMC7270359; doi:10.3389/fncel.2020.00113)
Supplement: FIGURE S1 — Sequence alignment of green and red fluorescent proteins. Homologous residues are color coded, whereas homology shared by all proteins is highlighted in yellow. Stars indicate the chromophore. Isoleucine residue in mPlum is indicated by blue circle. The T203 position (GFP numbering) is indicated by black arrowhead. Note that several PA-RFPs share positively charged amino acids (H or R) at the corresponding T203 position. [file Image_1.pdf]

Hussein and Berlin Suppl.1

```
                                                                                      ***
eGFP            MVSKGEEL----FTGVVPILVELDGDVNGHKFSVSGEGEGDATYGKLTLKFIC-TTGKLPVPWPTLVTTLTYGVQCFSRYPDHMKQHDFFKSAMPEGYVQ
PA-GFP          MVSKGEEL----FTGVVPILVELDGDVNGHKFSVSGEGEGDATYGKLTLKFIC-TTGKLPVPWPTLVTTFSYGVQCFSRYPDHMKQHDFFKSAMPEGYVQ
mcherry         MVSKGEEDNMAIIKEFMRFKVHMEGSVNGHEFEIEGEGEGRPYEGTQTAKLKVTKGGPLPFAWDILSPQFMYGSKAYVKHPADI--PDYLKLSFPEGFKW
PA-mcherry3     MVSKGEEDNMAIIKEFMRFKVHLEGSVNGHEFEIEGEGEGRPYEGTQTAKLKVTKGGPLPFTWDILSPQFMYGSNAYVKHPADI--PDYFKLSFPEGFKW
TagRFP          --------MSELIKENMHMKLYMEGTVNNHHFKCTSEGEGKPYEGTQTMRIKVVEGGPLPFAFDILATSFMYGSRTFINHTQGI--PDFFKQSFPEGFTW
PA-TagRFP       --------MSELIKENMHMKLYMEGTVNNHHFKCTSEGEGKPYEGTQTMRIKVVEGGPLPFAFDILATSFMYGSSTFINHTQGI--PDFFKQSWPEGFTW
PA-TagRFP1314   --------MSELIKENMHMKLYMEGTVNNHHFKCTSEGEGKPYEGTQTMRIKVVEGGPLPFAFDILATSFMYGSSTFINHTQGI--PDFWKQSFPEGFTW
PA-TagRFP1297   --------MSELIKENMHMKLYMEGTVNNHHFKCTSEGEGKPYEGTQTMRIKVVEGGPLPFAFDILATSFMYGSSTFINHTQGI--PDFWKQSFPEGFTW
mApple          MVSKGEENNMAIIKEFMRFKVHMEGSVNGHEFEIEGEGEGRPYEAFQTAKLKVTKGGPLPFAWDILSPQFMYGSKVYIKHPADI--PDYFKLSFPEGFRW
mPlum           MVSKGE----EVIKEFMRFKEHMEGSVNGHEFEIEGEGEGRPYEGTQTARLKVTKGGPLPFAWDILSPQIMYGSKAYVKHPADI--PDYLKLSFPEGFKW
mRuby           --------MNSLIKENMRMKVVLEGSVNGHQFKCTGEGEGNPYMGTQTMRIKVIEGGPLPFAFDILATSFMYGSRTFIKYPKGI--PDFFKQSFPEGFTW

eGFP            ERTIFFKDDGNYKTRAEVKFEGDTLVNRIELKGIDFKEDGNILGHKL-EYNYNSHNVYIMADKQKNGIKVNFKIRHNIEDGSV---QLADHYQQNTPIGD
PA-GFP          ERTIFFKDDGNYKTRAEVKFEGDTLVNRIELKGIDFKEDGNILGHKL-EYNYNSHNVYIMADKQKNGIKANFKIRHNIEDGSV---QLADHYQQNTPIGD
mcherry         ERVMNFEDGGVVTVTQDSSLQDGEFIYKVKLRGTNFPSDGPVMQKKTMGWEASSERMYPEDG----ALKGEIKQRLKLKDGGHYDAEVKTTYKAKKPV--
PA-mcherry3     ERVMNFEDGGVVTVTQDSSLQDGEFIYKVKLRGTNFPSDGPVIQKKTMGWDALSERMYPEDG----ALKGELKARLKLKDGGHYEAEVKTTYKAKKPV--
TagRFP          ERVTTYEDGGVLTATQDTSLQDGCLIYNVKIRGVNFPSNGPVMQKKTLGWEANTEMLYPADG----GLEGRSDMALKLVGGGHLICNFKTTYRSKKPAKN
PA-TagRFP       ERVTTYEDGGVLTATQDTSLQDGCLIYNVKIRGVNFPSNGPVMKKKTLGWEPSTEKLKPADG----GLEGRVDMALKLVGGGHLICNFKTTYRSKKPAKN
PA-TagRFP1314   ERVTTYEDGGVLTATQDTSLQDGCLIYNVKIRGVNFPSNGPVMKKKTLGWEPSTEMLYPADG----GLEGVVNMALKLVGGGHLICNFKTTYRSKKPAKN
PA-TagRFP1297   ERVTTYEDGGVLTATQDTSLQDGCLIYNVKIRGVNFPSNGPVMKKKTLGWEPSTEMLYPADG----GLEGVVNMALKLVGGGHLICNFKTTYRSKKPAKN
mApple          ERVMNFEDGGIIHVNQDSSLQDGVFIYKVKLRGTNFPSDGPVMQKKTMGWEASEERMYPEDG----ALKSEIKKRLKLKDGGHYAAEVKTTYKAKKPV--
mPlum           ERVMNFEDGGVVTVTQDSSLQDGEFIYKVKVRGTNFPSDGPVMQKKTMGWEASSERMYPEDG----ALKGEMKMRLRLKDGGHYDAEVKTTYMAKKPV--
mRuby           ERVTRYEDGGVITVMQDTSLEDGCLVYHAQVRGVNFPSNGAVMQKKTKGWEPNTEMMYPADG----GLRGYTHMALKVDGGGHLSCSFVTTYRSKKTVGN
                            ▼
eGFP            GPVLLPDNHYLSTQSALSKDPNEKRDHMVLLEFVTAAGITLGMDELYK--------------------------
PA-GFP          GPVLLPDNHYLSHQSKLSKDPNEKRDHMVLLEFVTAAGITLGMDELYK--------------------------
mcherry         ---QLPGAYNVNIKLDITS-HNEDYTIVEQYERAEGRHSTGGMDELYK--------------------------
PA-mcherry3     ---QLPGAYNVNRKLDITS-HNEDYTIVEQYERAEGRHSTGGMDELYK--------------------------
TagRFP          --LKMPGVYYVDHRLERIK-EADKETYVEQHEVAVARYCDLPS------KLGHK--------------------
PA-TagRFP       --LKMPGVYYVDRRLEIIK-EADKETYWEQHEVAVARYSDLPS------KLGHK--------------------
PA-TagRFP1314   --LKMPGVYYVDRRLEQIK-EADKETYWEQHEVAVARYCDLPS------KLGHK--------------------
PA-TagRFP1297   --LKMPGVYYVDRRLEQIK-EADKETYWEQHEVAVARYCDLPS------KLGHK--------------------
mApple          ---QLPGAYIVDIKLDIVS-HNEDYTIVEQYERAEGRHSTGGMDELYKSGLRSRAQASNSAVDGTAGPGSTGSR
mPlum           ---QLPGAYKTDIKLDITS-HNEDYTIVEQYERAEGRHSTGA--------------------------------
mRuby           --IKMPGIHAVDHRLERLE-ESDNEMFVVQREHAVAKFAGLGG------G------------------------
```
