# Supplementary figures and images for: Red Photoactivatable Genetic Optical-Indicators
Source: Front Cell Neurosci. 2020 May 28;14:113. doi: 10.3389/fncel.2020.00113 (PMC7270359; doi:10.3389/fncel.2020.00113)

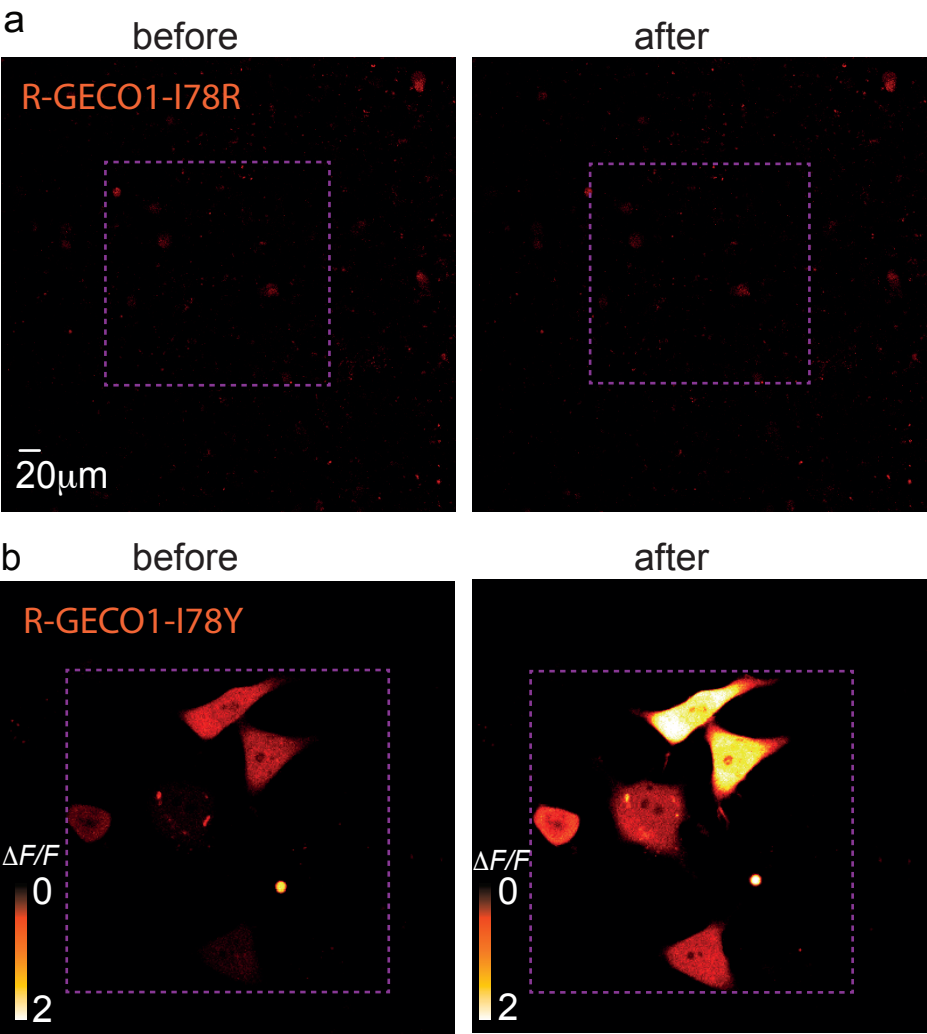

Supplement: FIGURE S2 — R-GECO1R and R-GECO1Y. (a) R-GECO1R does not express nor undergoes photoactivation in HeLa cells. Micrographs show HeLa cell before (left) and after (right) photoactivation (magenta dashed square). (b) R-GECO1Y undergoes photoactivation in HeLa cells. Micrographs show HeLa cell before (left) and after (right) photoactivation (magenta dashed square). [file Image_2.pdf]

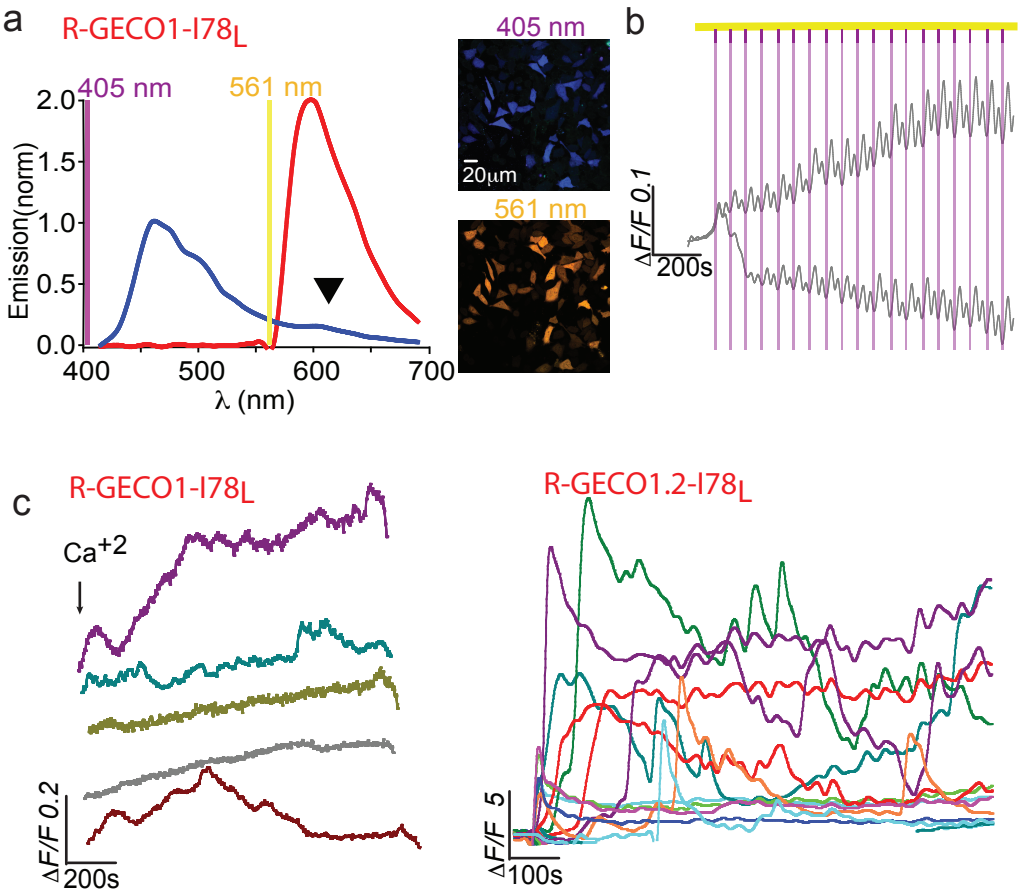

Supplement: FIGURE S3 — R-GECO1L retains light-induced artifacts. (a) Emission spectra of R-GECO1L collected from HeLa cells before photoactivation by use of 405 nm (blue plot and top micrograph) or 561 nm (red plot and bottom micrograph). Arrowhead indicates the weak direct excitation of the red chromophore by 405 nm. (b) R-GECO1L does not undergo photoactivation in HeLa cells. Cells imaged by 561 nm (yellow bar) are intermittently excited by short (1 s) bouts of 405 nm (magenta bars) does not exhibit stable increases in Ca2+-independent fluorescence but exhibits light-induced artifacts. (c) R-GECO1L does not responding to Ca2+ (left), whereas R-GECO1.2L does (right). [file Image_3.pdf]

a

R-GECO1-I78T

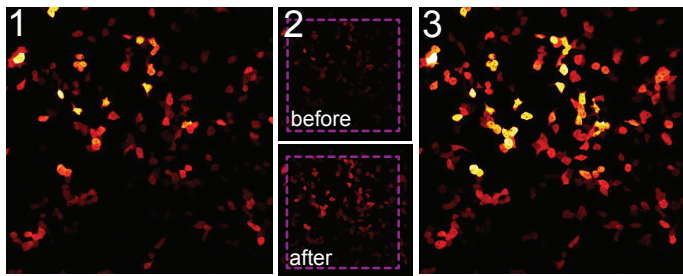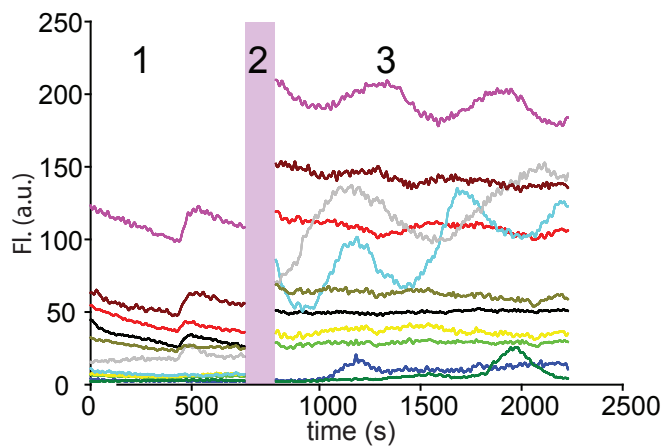

b

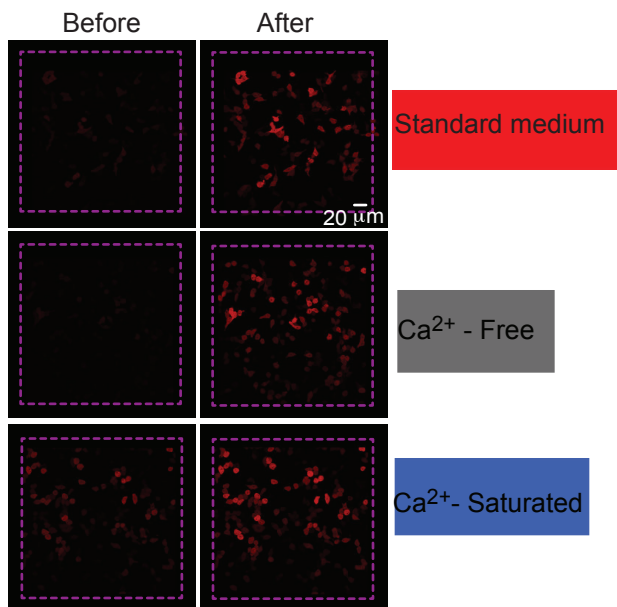

c

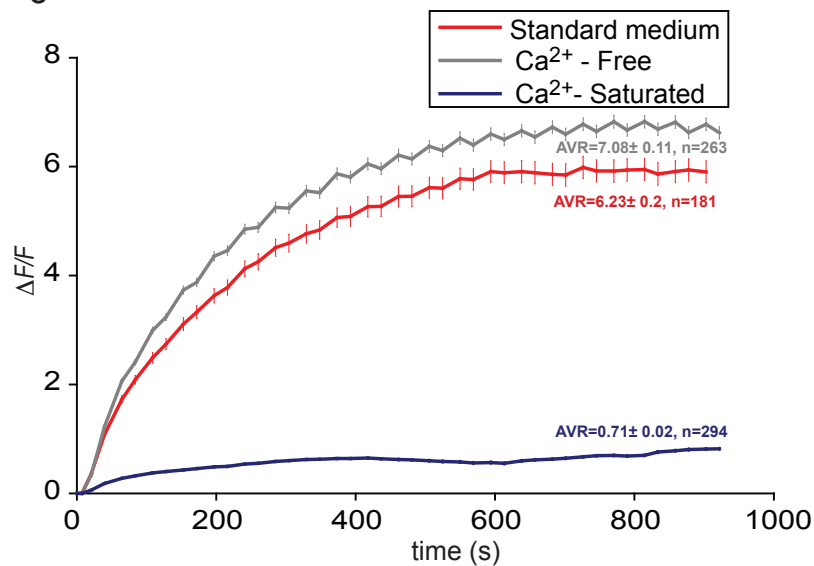

d

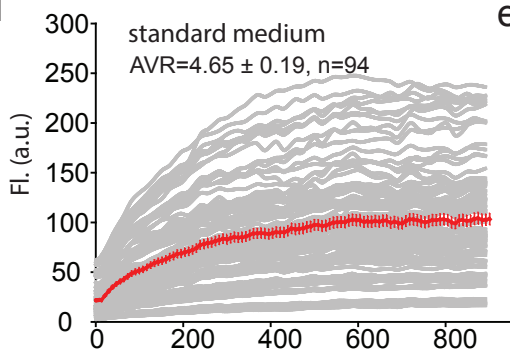

e

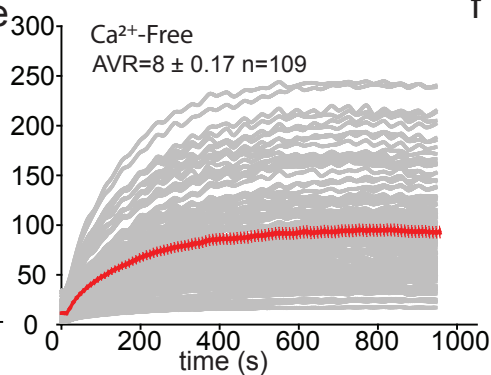

f

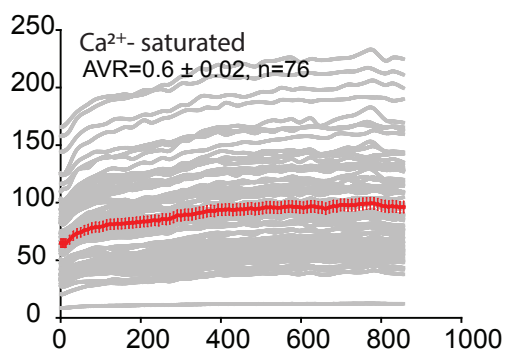

g

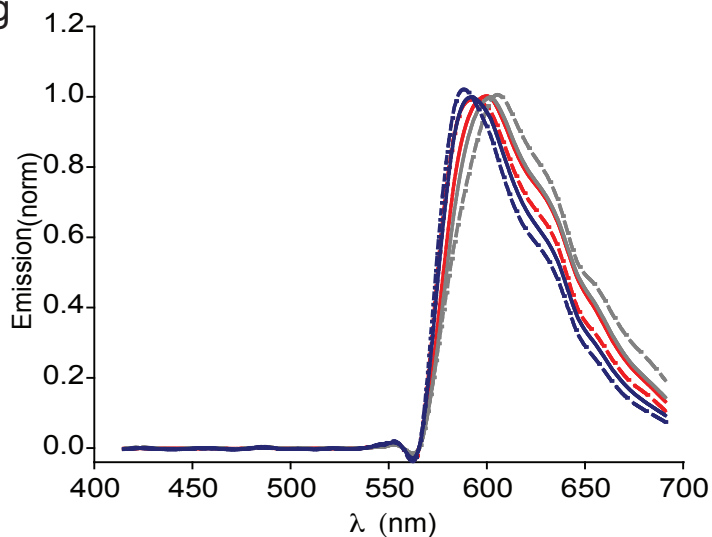

h

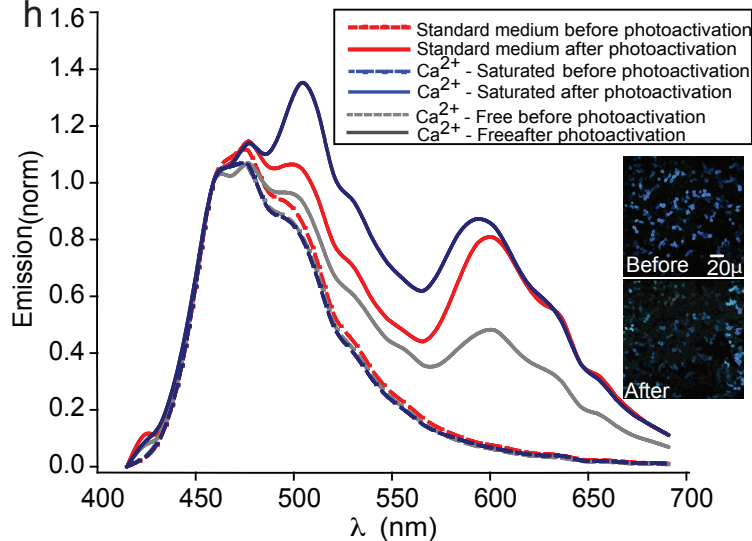

Supplement: FIGURE S4 — Characterization of the effect of Ca2+ on photoactivation and emission spectra of R-GECO1-I78T. (a) Ca2+-activity before and after photoactivation. Left: micrographs of R-GECO1-I78T expressed in HEK293 cells; initially (1), during photoactivation (2; before and after), and after photoactivation (3). Data are summarized in plot (right). Note the large increase in the amplitudes of the spontaneous Ca2+-oscillations after photoactivation (magenta bar). (b,c) Extent of photoactivation of R-GECO1-I78T under different conditions. (b) Micrographs of R-GECO1-I78T expressed in HEK293 cells in standard medium (top, red), Ca2+-free (middle, gray) and Ca2+-saturated (bottom, dark blue) conditions, before and after photoactivation. Data are summarized in c. Note that Ca2+-free and standard conditions are beneficial to photoactivation, whereas the presence of Ca2+ is detrimental. (d–f) Representative experiment showing raw fluorescence traces before and after photoactivation of HEK293 cells in different meda (as summarized in c). These show the detectable basal fluorescence (above zero) from which we could faithfully calculate ΔF/F (see section “Materials and Methods”). Traces also show that the baseline drastically changes, though maximal fluorescence is obtained under all conditions. Emission spectra induced by 561 nm illumination (g) or 405 nm (h), under different conditions (color coded as in e). [file Image_4.pdf]

R-GECO 1.2 I78T

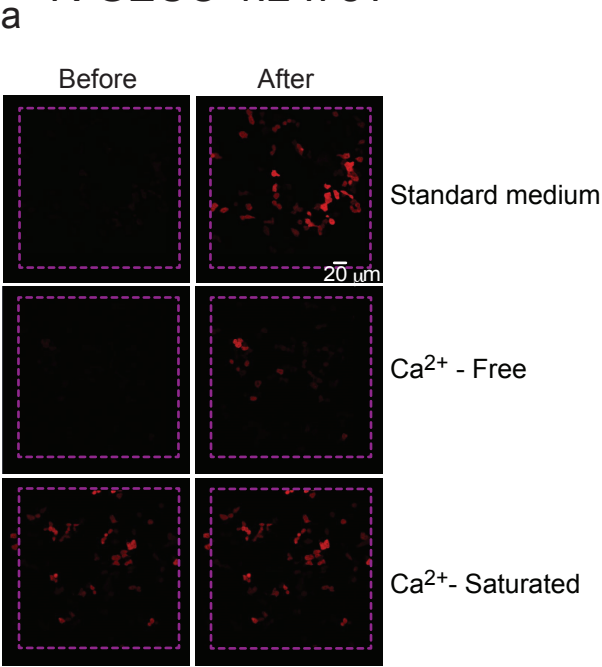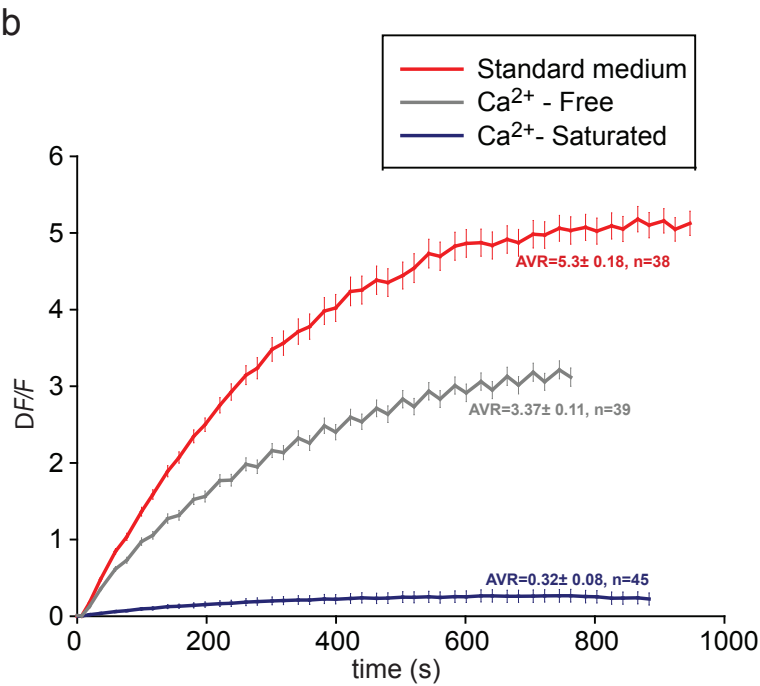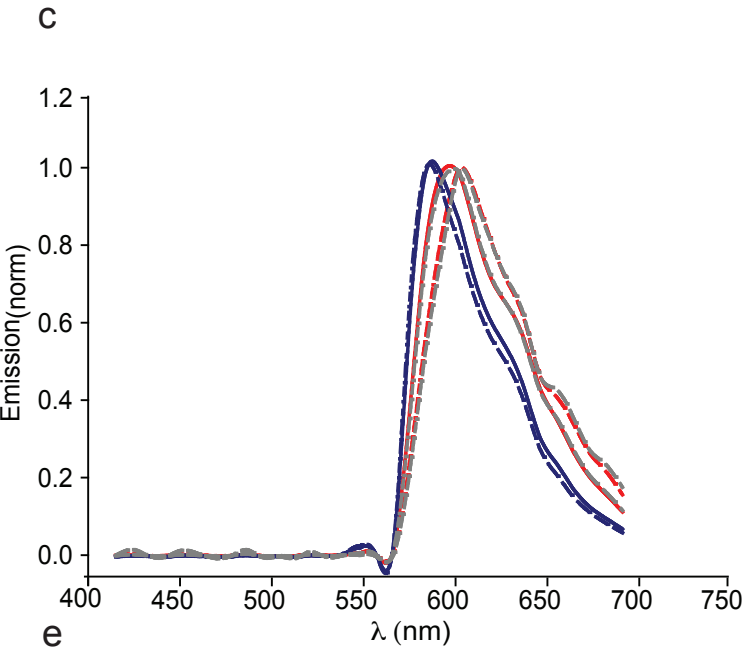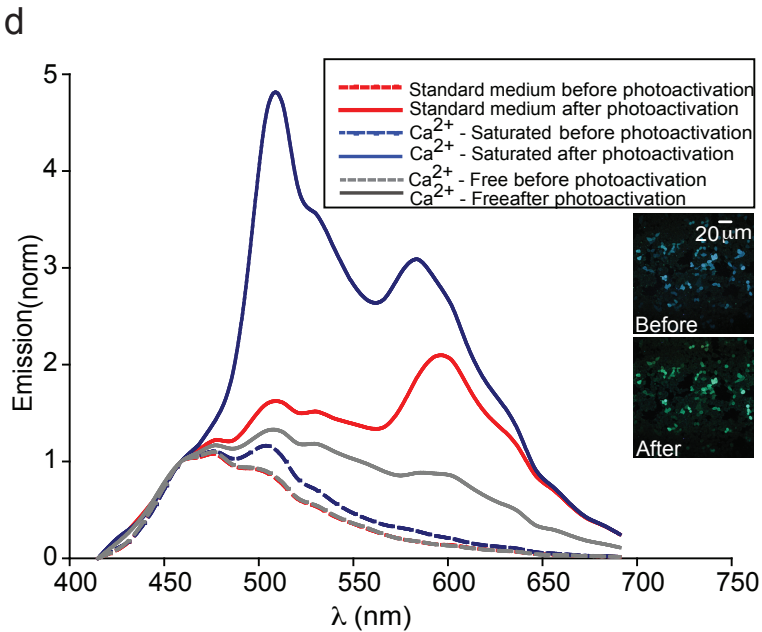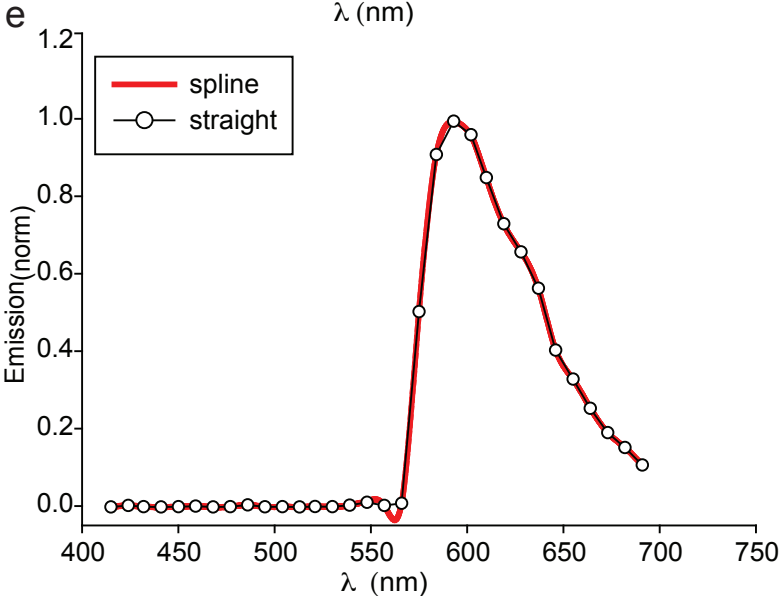

Supplement: FIGURE S5 — Characterization of the effect of Ca2+ on photoactivation and emission spectra of R-GECO1.2-I78T. (a) Micrographs of R-GECO1.2-I78T expressed in HEK293 cells in standard medium (top, red), Ca2+-free (middle, gray), and Ca2+-saturated (bottom, dark blue) conditions, before and after photoactivation. (b) Extent of photoactivation of R-GECO1.2-I78T under different conditions (color coded as in a). Note that Ca2+-free and standard conditions are beneficial to photoactivation, whereas the presence of Ca2+ is detrimental. Emission spectra induced by 561 nm illumination (c) or 405 nm (d), under different conditions (color coded as in a). Note the striking increase in the emission of 512 nm. (e) Comparison between straight and smoothed lines for plotting emission spectra. A representative emission spectrum induced by 561 nm. Data points are collected every 8 nm (open circles; see section “Materials and Methods”). Plot is constructed by straight lines (black) or smoothed (splined, red plot). Note the agreement between both plots. [file Image_5.pdf]

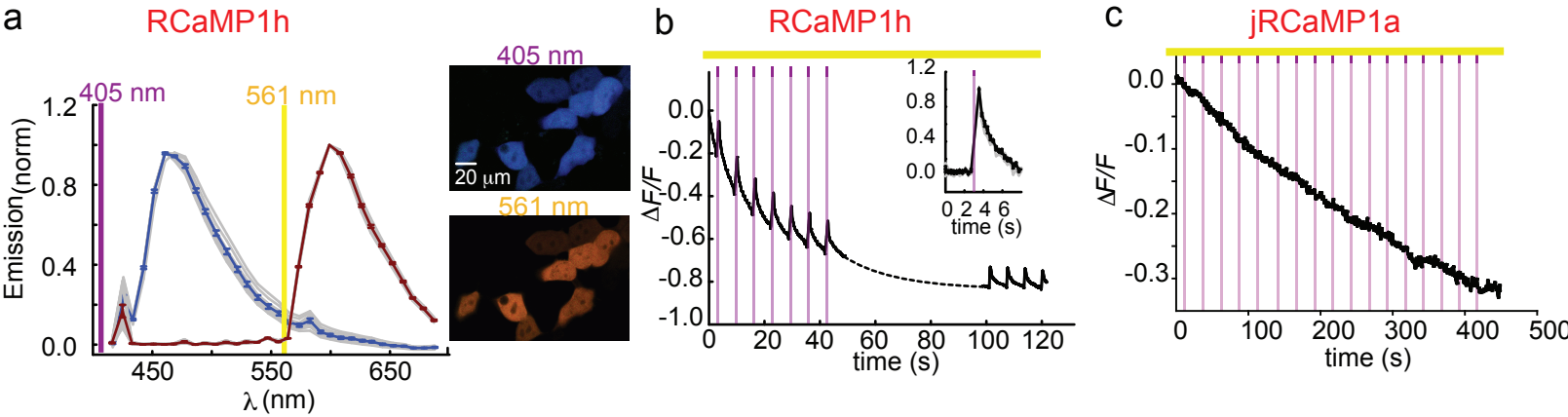

Supplement: FIGURE S6 — Characterization of RCaMP1h and JRCaMP1b. (a) Emission spectra of RCaMP1h collected from HeLa cells before photoactivation by use of 405 nm (blue plot and top micrograph) or 561 nm (red plot and bottom micrograph). RCaMP1h (b) exhibits transient photoconversion during intervals of near UV light irradiation (405 nm), whereas jRCaMP1a (c) does not, rather undergoes slight photobleaching. [file Image_6.pdf]

**a**  
jRCaMP1a H134I

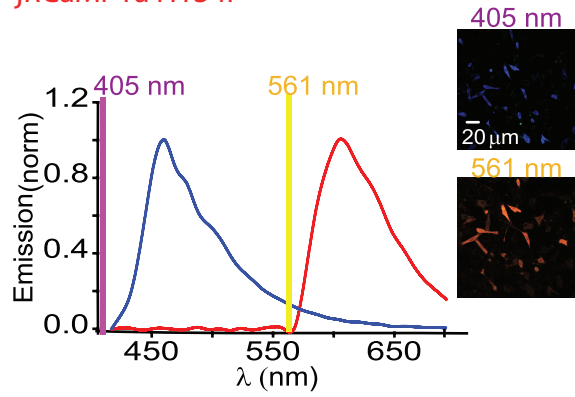

**b**  
jRCaMP1a H134T

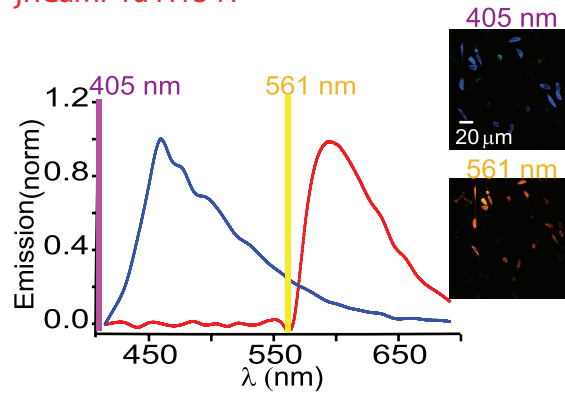

**c**  
jRCaMP1a H134I

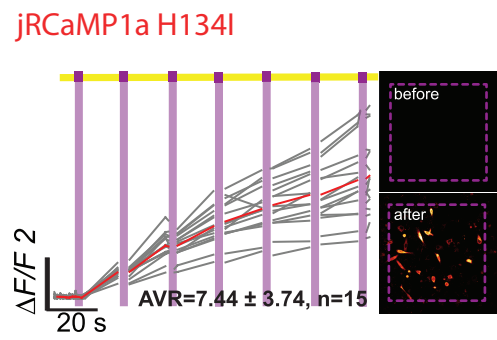

**d**  
jRCaMP1a H134T

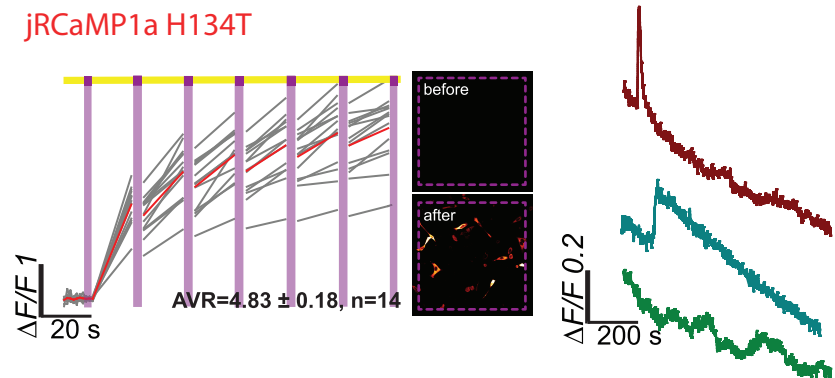

**e**  
jRCaMP1b H134I

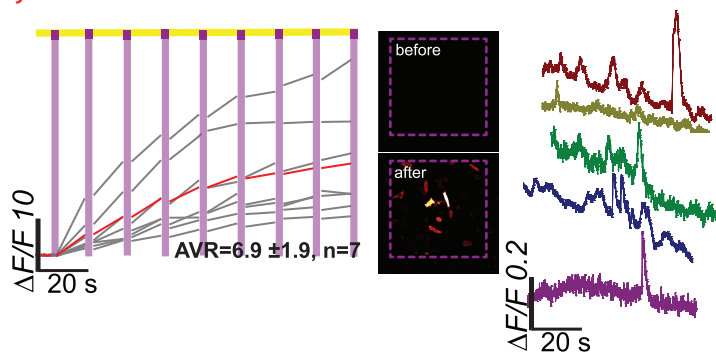

**f**  
jRCaMP1b H134T

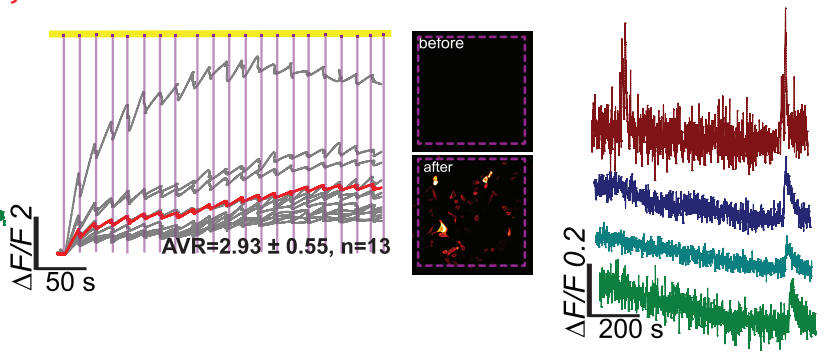

**g**

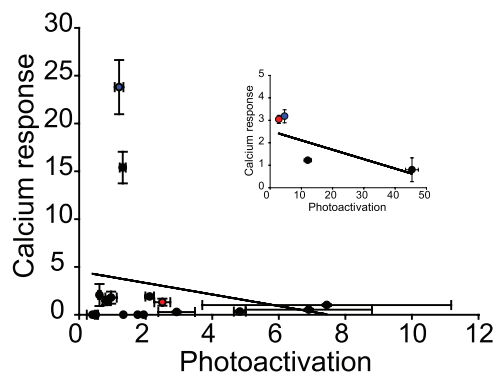

Supplement: FIGURE S7 — Photoactivatable JRCaMP1a and JRCaMP1b are poor Ca2+-probes. Emission spectra of JRCaMP1aI (a) and JRCaMP1aT (b) collected from HeLa cells before photoactivation by use of 405 nm (blue plots and top micrographs) or 561 nm (red plots and bottom micrographs). Stable photoactivation of JRCaMP1aI (c), JRCaMP1aT (d), JRCaMP1bI (e), or JRCaMP1bT (f) in HeLa cells. Cells imaged by 561 nm (yellow bar) are intermittently excited by short (1 s) bouts of 405 nm (magenta bars) exhibit stable increase in Ca2+-independent fluorescence. Micrographs show HeLa cells imaged by 561 nm before (top) and after (bottom) photoactivation (magenta dashed square). (Right) Representative traces showing Ca2+-activity following the application of Ca2+ (+50 μM) of JRCaMP1aI (c), JRCaMP1aT (d), JRCaMP1bI (e), and JRCaMP1bT (f). (g) Correlation between extent of photoactivation and Ca2+-performance of various probes in HeLa cells and in HEK293 cells (inset). R-GECO1-I78T (red) and R-GECO1.2-I78T (blue) are highlighted. Note the inverse trend between the features (though non-significant, Spearman correlation, see section “Materials and Methods”). [file Image_7.pdf]
