# Supplementary material for: Red Photoactivatable Genetic Optical-Indicators
Source: Front Cell Neurosci. 2020 May 28;14:113. doi: 10.3389/fncel.2020.00113 (PMC7270359; doi:10.3389/fncel.2020.00113)
Supplement: TABLE S1 — List of primers used to generate the various mutants tested. Mutated residues are highlited (yellow). [file Table_1.docx]

| **R-GECO 1 and 1.2** | Mutation |  | Primer Sequence 5’->3’ (Forward- F), (Reverse-R) |
| --- | --- | --- | --- |
|  | I78L | F  R | CCCGGCGCCTACATCGTCGACCTTAAGTTGGACATCGTGTCCCAC  GTGGGACACGATGTCCAACTTAAGGTCGACGATGTAGGCGCCGGG |
|  | I78T | F  R | CCCGGCGCCTACATCGTCGACACAAAGTTGGACATCGTGTCCCAC  GTGGGACACGATGTCCAACTTTGTGTCGACGATGTAGGCGCCGGG |
|  | I78N | F  R | CCCGGCGCCTACATCGTCGACAACAAGTTGGACATCGTGTCCCAC  GTGGGACACGATGTCCAACTTGTTGTCGACGATGTAGGCGCCGGG |
|  | I78H | F  R | CCCGGCGCCTACATCGTCGACCATAAGTTGGACATCGTGTCCCAC  GTGGGACACGATGTCCAACTTATGGTCGACGATGTAGGCGCCGGG |
|  | I78A | F  R | CCCGGCGCCTACATCGTCGACGCTAAGTTGGACATCGTGTCCCAC  GTGGGACACGATGTCCAACTTAGCGTCGACGATGTAGGCGCCGGG |
|  | I78Y | F  R | CCCGGCGCCTACATCGTCGACTATAAGTTGGACATCGTGTCCCAC  GTGGGACACGATGTCCAACTTATAGTCGACGATGTAGGCGCCGGG |
|  | I78Q | F  R | CCCGGCGCCTACATCGTCGACCAAAAGTTGGACATCGTGTCCCAC  GTGGGACACGATGTCCAACTTTTGGTCGACGATGTAGGCGCCGGG |
|  | I78PRO | F  R | CCCGGCGCCTACATCGTCGACCCTAAGTTGGACATCGTGTCCCAC  GTGGGACACGATGTCCAACTTAGGGTCGACGATGTAGGCGCCGGG |
|  | I78Val | F  R | CCCGGCGCCTACATCGTCGACGTTAAGTTGGACATCGTGTCCCAC  GTGGGACACGATGTCCAACTTAACGTCGACGATGTAGGCGCCGGG |
|  | I78C | F  R | CCCGGCGCCTACATCGTCGACTGTAAGTTGGACATCGTGTCCCAC  GTGGGACACGATGTCCAACTTACAGTCGACGATGTAGGCGCCGGG |
|  | I78K | F  R | CCCGGCGCCTACATCGTCGACAAAAAGTTGGACATCGTGTCCCAC  GTGGGACACGATGTCCAACTTTTTGTCGACGATGTAGGCGCCGGG |
|  | I78G | F  R | CCCGGCGCCTACATCGTCGACGGTAAGTTGGACATCGTGTCCCAC  GTGGGACACGATGTCCAACTTACCGTCGACGATGTAGGCGCCGGG |
|  | I78F | F  R | CCCGGCGCCTACATCGTCGACTTTAAGTTGGACATCGTGTCCCAC  GTGGGACACGATGTCCAACTTAAAGTCGACGATGTAGGCGCCGGG |
|  | I78D | F  R | CCCGGCGCCTACATCGTCGACGATAAGTTGGACATCGTGTCCCAC  GTGGGACACGATGTCCAACTTATCGTCGACGATGTAGGCGCCGGG |
|  | I78E | F  R | CCCGGCGCCTACATCGTCGACGAAAAGTTGGACATCGTGTCCCAC  GTGGGACACGATGTCCAACTTTTCGTCGACGATGTAGGCGCCGGG |
|  | I78S | F  R | CCCGGCGCCTACATCGTCGACTCTAAGTTGGACATCGTGTCCCAC  GTGGGACACGATGTCCAACTTAGAGTCGACGATGTAGGCGCCGGG |
|  | I78R | F  R | CCCGGCGCCTACATCGTCGACCGTAAGTTGGACATCGTGTCCCAC  GTGGGACACGATGTCCAACTTACGGTCGACGATGTAGGCGCCGGG |
|  | I78W | F  R | CCCGGCGCCTACATCGTCGACTGGAAGTTGGACATCGTGTCCCAC  GTGGGACACGATGTCCAACTTCCAGTCGACGATGTAGGCGCCGGG |
| **mRuby3** | H200I | F  R | CCCGGTGTCCATGCCGTTGATATCCGCCTGGAAAGGATCGAGGAG  CTCCTCGATCCTTTCCAGGCGGATATCAACGGCATGGACACCGGG |
|  | H200T | F  R | CCCGGTGTCCATGCCGTTGATACACGCCTGGAAAGGATCGAGGAG  CTCCTCGATCCTTTCCAGGCGTGTATCAACGGCATGGACACCGGG |
| **RCaMP1h**  **(mRuby)** | H115T | F  R | CCTGCCATTCATTACGTCAGCACACGTCTGGAGCGCCTGGAGGAG  CTCCTCCAGGCGCTCCAGACGTGTGCTGACGTAATGAATGGCAGG |
| **jRCaMP1a**  **(mRuby)** | H134I | F  R | CCTGCCATTCATAGCGTCAGCATTCGTCTGGAGCGCCTGGAGGAG  CTCCTCCAGGCGCTCCAGACGAATGCTGACGCTATGAATGGCAGG |
|  | H134T | F  R | CCTGCCATTCATAGCGTCAGCACTCGTCTGGAGCGCCTGGAGGAG  CTCCTCCAGGCGCTCCAGACGAGTGCTGACGCTATGAATGGCAGG |
| **jRCaMP1b**  **(mRuby)** | H134I | F  R | CCTGCCATTCATTACGTCAGCATCCGTCTGGAGCGCCTGGAGGAG  CTCCTCCAGGCGCTCCAGACGGATGCTGACGTAATGAATGGCAGG |
|  | H134T | F  R | CCTGCCATTCATTACGTCAGCACACGTCTGGAGCGCCTGGAGGAG  CTCCTCCAGGCGCTCCAGACGTGTGCTGACGTAATGAATGGCAGG |
|  | H134K | F  R | CCTGCCATTCATTACGTCAGCAAGCGTCTGGAGCGCCTGGAGGAG  CTCCTCCAGGCGCTCCAGACGCTTGCTGACGTAATGAATGGCAGG |
|  | H134R | F  R | CCTGCCATTCATTACGTCAGCCGTCGTCTGGAGCGCCTGGAGGAG  CTCCTCCAGGCGCTCCAGACGACGGCTGACGTAATGAATGGCAGG |
| **jRGECOa**  **(mApple)** | I131T | F  R | CCCGGCGCCTACATCGTGGACACCAAGTTGGACATCGTGTCCCAC  GTGGGACACGATGTCCAACTTGGTGTCCACGATGTAGGCGCCGGG |
|  | I131H | F  R | CCCGGCGCCTACATCGTGGACCACAAGTTGGACATCGTGTCCCAC  GTGGGACACGATGTCCAACTTGTGGTCCACGATGTAGGCGCCGGG |
|  | I131R | F  R | CCCGGCGCCTACATCGTGGACAGGAAGTTGGACATCGTGTCCCAC GTGGGACACGATGTCCAACTTCCTGTCCACGATGTAGGCGCCGGG |
